# One‐Step Formed Janus Hydrogel with Time‐Space Regulating Properties for Suture‐Free and High‐Quality Tendon Healing

**DOI:** 10.1002/advs.202411400

**Published:** 2025-02-08

**Authors:** Chenguang Ouyang, Tian Tu, Haojie Yu, Li Wang, Zhipeng Ni, Jian Yang, Yanzhao Dong, Xiaodi Zou, Weijie Zhou, Jinyi Liu, Dingning Chen, Yu Wang, Xudong Wu, Hong Yi, Xunchun Yuan, Zhenfeng Liu, Hui Lu

**Affiliations:** ^1^ State Key Laboratory of Chemical Engineering College of Chemical and Biological Engineering Zhejiang University Hangzhou Zhejiang 310058 China; ^2^ Department of plastic and aesthetic The First Affiliated Hospital College of Medicine Zhejiang University Hangzhou Zhejiang 310003 China; ^3^ Department of Orthopedics The First Affiliated Hospital College of Medicine Zhejiang University Hangzhou Zhejiang 310003 China; ^4^ Department of Nuclear Medicine The First Affiliated Hospital College of Medicine Zhejiang University Hangzhou Zhejiang 310003 China

**Keywords:** janus hydrogel, one‐step synthesis, time‐space regulation, tendon healing, wet adhesion

## Abstract

Janus hydrogels have promising applications in tendon healing and anti‐peritendinous adhesions. However, their complicated preparation methods, weak mechanical properties, and unstable adhesion interfaces have severely limited their application in suture‐free and high‐quality tendon healing. In this work, by controlling the interfacial distribution of free ‐COOH groups and cationic‐π structures on both sides of the hydrogels, a series of PZBA‐EGCG‐ALC Janus hydrogels with varying degrees of asymmetric properties are successfully prepared using a simple and efficient one‐step synthesis method. The tensile strength and elongation at the break of the Janus hydrogel are as high as 0.51 ± 0.04 MPa and 922.89 ± 28.59%. In addition, the Janus hydrogel can achieve a high difference in adhesion strength (nearly 20‐fold) while maintaining a strong adhesion strength on their bottom sides (up to 524.8 ± 33.1 J m^−2^). In the spatial dimension, its excellent mechanical compliance and one‐sided adhesion behavior can provide effective mechanical support and physical barriers for the injured Achilles tendons. More importantly, the Janus hydrogel can also minimize early inflammation generation in the time dimension via its ROS‐responsive PZBA‐EGCG prodrug macromolecules. This study provided a more effective and convenient suture‐free strategy for constructing Janus hydrogels to promote high‐quality tendon healing.

## Graphical abstract

This study establishes a simple and efficient one‐step synthesis method to prepare Janus hydrogels for suture‐free and high‐quality tendon healing. The Janus hydrogels with time‐space regulating properties can minimize early tendon inflammation to promote tendon healing, while providing effective mechanical support and physical barriers for injured tendons to prevent postoperative adhesions.

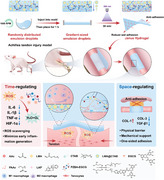

## 1 Introduction

Tendons are tough, band‐like structures that consist of fibrous, viscoelastic connective tissue.^[^ ^1^, ^2^ ^]^ They anchor muscles to bones and play a vital role in resisting tension and aiding movement.^[^ ^1^, ^2^ ^]^ Tendon injuries tend to occur in the elderly population as well as in athletes and others who engage in high mechanical loads and weight‐bearing activities.^[^ ^3^ ^]^ It is believed that more than 30 million people worldwide are affected by tendon injuries, with an annual medical budget of more than $140 billion.^[^ ^4^, ^5^ ^]^ Up to now, surgical suturing remains the gold standard for repairing tendon ruptures.^[^ ^2^, ^6^ ^]^ However, during tendon regeneration, due to its low cellular and vascular density and limited self‐regeneration capacity, local inflammation caused by early oxidative stress injury often occurs.^[^ ^7^ ^]^ They are likely to induce postoperative scar formation as well as peritendinous adhesions, which significantly weaken the biomechanical properties of the repaired tendon and increase the risk of re‐rupture.^[^ ^8^, ^9^, ^10^ ^]^ After Achilles tendon surgery, although systemic and in situ application of corticosteroids and NSAIDs can effectively reduce the inflammatory response and alleviate postoperative pain, the side effects associated with prolonged administration of the drugs should not be ignored.^[^ ^11^, ^12^, ^13^ ^]^ Therefore, in order to temporally and spatially provide localized mechanical support and targeted drug delivery to postoperative tendon tissues, new biomaterial‐based therapies are urgently needed to be developed.

Considering the time dimension, surgically repaired tendon healing goes through three overlapping phases, i.e., inflammation (days 1–7), proliferation (days 3–14), and remodeling (days 10 onward).^[^ ^14^, ^15^ ^]^ Among these, the early inflammatory response is closely related to the formation of fibrous adhesions.^[^ ^15^, ^16^, ^17^ ^]^ By utilizing ROS‐responsive materials and linkers, targeted drug delivery systems have been developed to improve the uncontrolled drug release behavior and effectively avoid its undesired diffusion in the postoperative Achilles tendon region.^[^ ^18^, ^19^ ^]^ Considering the spatial dimension, biomaterials are expected to match with tendon biomechanics and possess asymmetric tissue adhesion properties.^[^ ^9^ ^]^ Specifically, the side adjacent to the injured Achilles tendon should have tough adhesion and excellent bioactivity to provide mechanical support and a favorable microenvironment for tendon repair. However, the other side should preferably be non‐adhesive to the surrounding tissue to minimize friction and thus physically prevent fibrotic scar formation. To meet these needs, hydrogels have become an ideal option for suture‐free and high‐quality tendon healing due to their intrinsic physiochemical similarity to biological tissues. Through bridging chitosan molecules on the surface of a dissipative alginate acrylamide hydrogel and loading the drug triamcinolone acetonide, Mooney et al. achieved mechanical tissue integrity and controlled spatiotemporal drug delivery for tendon injuries.^[^ ^20^ ^]^ Similarly, Yao et al. developed a dual dynamic cross‐linked hyaluronic acid‐based hydrogel patch.^[^ ^21^ ^]^ Benefiting from the natural anti‐inflammatory and antimicrobial properties of protocatechuic aldehyde (PA), the hydrogel regulated macrophage polarization, eliminated oxidative stress, and reduced inflammation through NF‐κB signaling. It also synergized with the peripheral electrospun membrane to promote tendon healing in the spatial dimension. Our previous work designed a series of time‐space regulating nanohybrid prodrugs (tannic acid (TA), epigallocatechin (EGC), and epigallocatechin gallate (EGCG)) polyphosphazene hydrogels (PZBA‐TA/EGC/EGCG‐GG hydrogels) for preventing peritendinous adhesions.^[^ ^22^ ^]^ Although the time‐space regulating strategy has been shown to be effective in alleviating rat peritendinous adhesions, the hydrogel showed limited mechanical strength as well as indiscriminate two‐sided adhesion, often requiring additional peripherally lubricated hydrogel matrix/electrospun membranes to meet the requirements in the spatial dimension. After injecting or implanting the hydrogels, physical deformations caused by movement can lead to their displacement and mechanical fragmentation. There is also a risk of inducing severe postoperative adhesions between the tendon and the normal tissue. In recent years, in order to solve the above problems, Janus hydrogels have offered another possibility.^[^ ^9^, ^20^, ^23^ ^]^ However, most existing Janus hydrogels were obtained by stepwise composite processing^[^ ^24^, ^25^, ^26^, ^27^, ^28^, ^29^ ^]^ and unilateral ion sealing^[^ ^30^, ^31^, ^32^, ^33^ ^]^ approaches. These hydrogels suffer from the problems of time‐consuming preparation process, poor binding between different layers, mismatch in mechanical strength, and undesirable diffusion caused by drug loading. Therefore, there is an urgent need to develop a Janus hydrogel prepared by a simple and efficient one‐step synthesis approach, which exhibits a high degree of asymmetric adhesion to tissues while maintaining tough mechanical strength to prevent postoperative peritendinous adhesions.^[^ ^34^, ^35^, ^36^, ^37^ ^]^

**Scheme 1:**
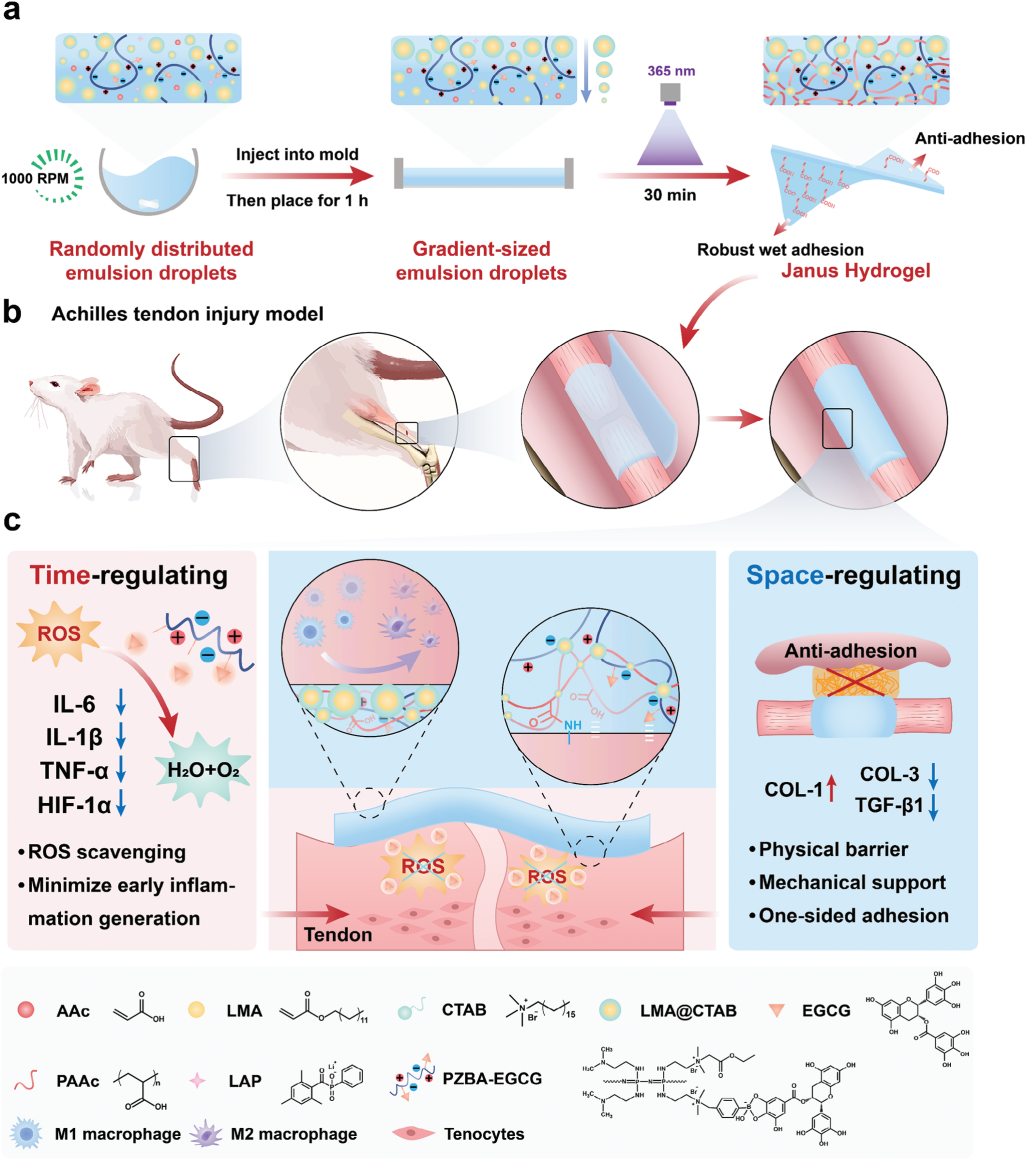
Schematic design and preparation of the one‐step formed Janus hydrogel with time‐space regulating properties for suture‐free and high‐quality tendon healing. a) Schematic flow diagram for the preparation of PZBA‐EGCG‐ALC Janus hydrogels. b) Schematic illustration of the PZBA‐EGCG‐ALC Janus hydrogels for the rat Achilles tendon injury model. c) Time‐space regulation mechanism of the PZBA‐EGCG‐ALC Janus hydrogels in promoting high‐quality tendon healing and preventing postoperative adhesions.

In this work, a series of PZBA‐EGCG‐ALC Janus hydrogels with varying degrees of asymmetric properties were successfully prepared using a simple and efficient one‐step synthesis method for promoting high‐quality tendon healing and preventing postoperative peritendinous adhesions (**Scheme** **1**a–c). Randomly distributed emulsion droplets (LMA@CTAB) were obtained in hydrogel precursor emulsions by stirring at a certain speed (e.g., 1000 RPM). After injecting the hydrogel precursor emulsions into the molds and placing them for 1 h, the LMA@CTAB emulsion droplets would form a gradient arrangement with decreasing sizes from top to bottom due to their buoyancy difference. Subsequently, PZBA‐EGCG‐ALC Janus hydrogels with asymmetric adhesion properties were obtained by UV photocuring. Due to the gradient barrier effect of LMA@CTAB emulsion droplets, the ‐COOH groups in PAAc and cationic‐π structures in PZBA‐ECCG prodrug macromolecular networks were exposed more to the bottom surface of the PZBA‐EGCG‐ALC Janus hydrogels, resulting in their asymmetric properties. It is worth noting that a series of PZBA‐EGCG‐ALC Janus hydrogels with varying degrees of asymmetric properties could be achieved by simply regulating the stirring speeds of the hydrogel precursor emulsions (i.e., 500 RPM–1000 RPM). And the PZBA‐EGCG‐ALC Janus hydrogels could achieve the difference in adhesion strength between the two sides from 1.6 to 19.7 folds while maintaining a strong adhesion strength on their bottom sides (>400 J m^−2^). More importantly, in the rat tendon injury model, compared with surgical sutures, the PZBA‐EGCG‐ALC Janus hydrogels exhibited several advantages in promoting tendon healing while effectively preventing postoperative peritendinous adhesions. In the time dimension, the PZBA‐EGCG prodrug macromolecules in the hydrogel networks had ROS‐responsive and scavenging abilities, effectively reducing the generation of early inflammation around the injury sites. In the spatial dimension, the hydrogels showed excellent mechanical compliance and one‐sided adhesion behavior, providing effective mechanical support and physical barriers for the injured Achilles tendons. Overall, the one‐step formed PZBA‐EGCG‐ALC Janus hydrogels provided a more convenient suture‐free strategy for promoting high‐quality tendon healing and preventing postoperative adhesions. They are promising as a potential candidate material for life‐saving internal surgeries and show great potential for future clinical translation.

## 2 Results and Discussion

### 2.1 Preparation of the PZBA‐EGCG‐ALC Janus Hydrogels

The poly[(*N,N*‐dimethylethylenediamine)‐*g*‐(*N,N,N,N*‐dimethylaminoethyl‐*p*‐methylphenylboronic acid ammonium bromide‐*g*‐(*N,N,N,N*‐dimethylaminoethyl ethyl acetate ammonium bromide)] (PZBA) was synthesized according to our previous work.^[^ ^22^ ^]^ The synthesis route is shown in Figure S1 (Supporting Information). The chemical structure of PZBA was examined by ^1^H NMR and ^31^P NMR (Figure S2, Supporting Information). Through the integration of the characteristic peak areas, the grafting ratios of tertiary amine moieties, phenylboronic acid moieties, and ethyl bromoacetate moieties were calculated to be 21.8%, 39.0%, and 39.2%, respectively. Only a single peak appeared in the ^31^P NMR spectrum of PZBA, proving that no side reactions such as hydrolysis and rearrangement occurred during its synthesis. The PZBA‐EGCG‐ALC Janus hydrogels were prepared by a simple and efficient one‐step synthesis approach (Scheme 1a). In order to construct hydrogels with favorable mechanical compliance and asymmetric properties in the spatial dimension, acrylic acid (AAc) was used as the hydrophilic monomer due to its free carboxyl group. And lauryl methacrylate (LMA) containing long carbon chains and hexadecyl trimethyl ammonium bromide (CTAB) were used as hydrophobic monomers and surfactants, respectively. Meanwhile, the PZBA‐EGCG prodrug macromolecules were introduced to offer more electrostatic interactions and ROS response capabilities for the formed hydrogel networks in the time dimension. In aqueous solutions, CTAB could self‐assemble with LMA to form LMA@CTAB emulsion droplets. Initially, LMA@CTAB emulsion droplets with different randomly distributed sizes were obtained in hydrogel precursor emulsions at different stirring speeds (i.e., 500 RPM–1500 RPM). After injecting the hydrogel precursor emulsions into the molds and placing them for 1 h, the LMA@CTAB emulsion droplets would form a gradient arrangement with decreasing sizes from top to bottom due to their buoyancy difference. Subsequently, a series of PZBA‐EGCG‐ALC Janus hydrogels with varying degrees of asymmetric properties were obtained by UV photocuring in the molds (The resulting Janus hydrogels were named as PZBA‐EGCG*_x_*‐ALC‐*y*, where *x* denotes the concentration of the natural polyphenol EGCG in the hydrogels, and *y* denote the stirring speed during the preparation of hydrogel precursor emulsions). The obtained PZBA‐EGCG‐ALC Janus hydrogels were dialyzed in deionized water to remove unreacted monomers. The detailed preparation process and formulation (Table S1, Supporting Information) of PZBA‐EGCG‐ALC Janus hydrogels are shown in the Experimental Section part. Fourier transform infrared spectroscopy (FT‐IR) was used to monitor the photopolymerization process of the PZBA‐EGCG‐ALC Janus hydrogels. As shown in Figure S3 (Supporting Information), the double‐bond stretching vibration peaks of AAc and LMA at 1630 cm^−1^ completely disappeared, indicating that the polymerization reaction was completed and no monomer remained.

### 2.2 Asymmetric Mechanism of PZBA‐EGCG‐ALC Janus Hydrogels

**Figure 1:**
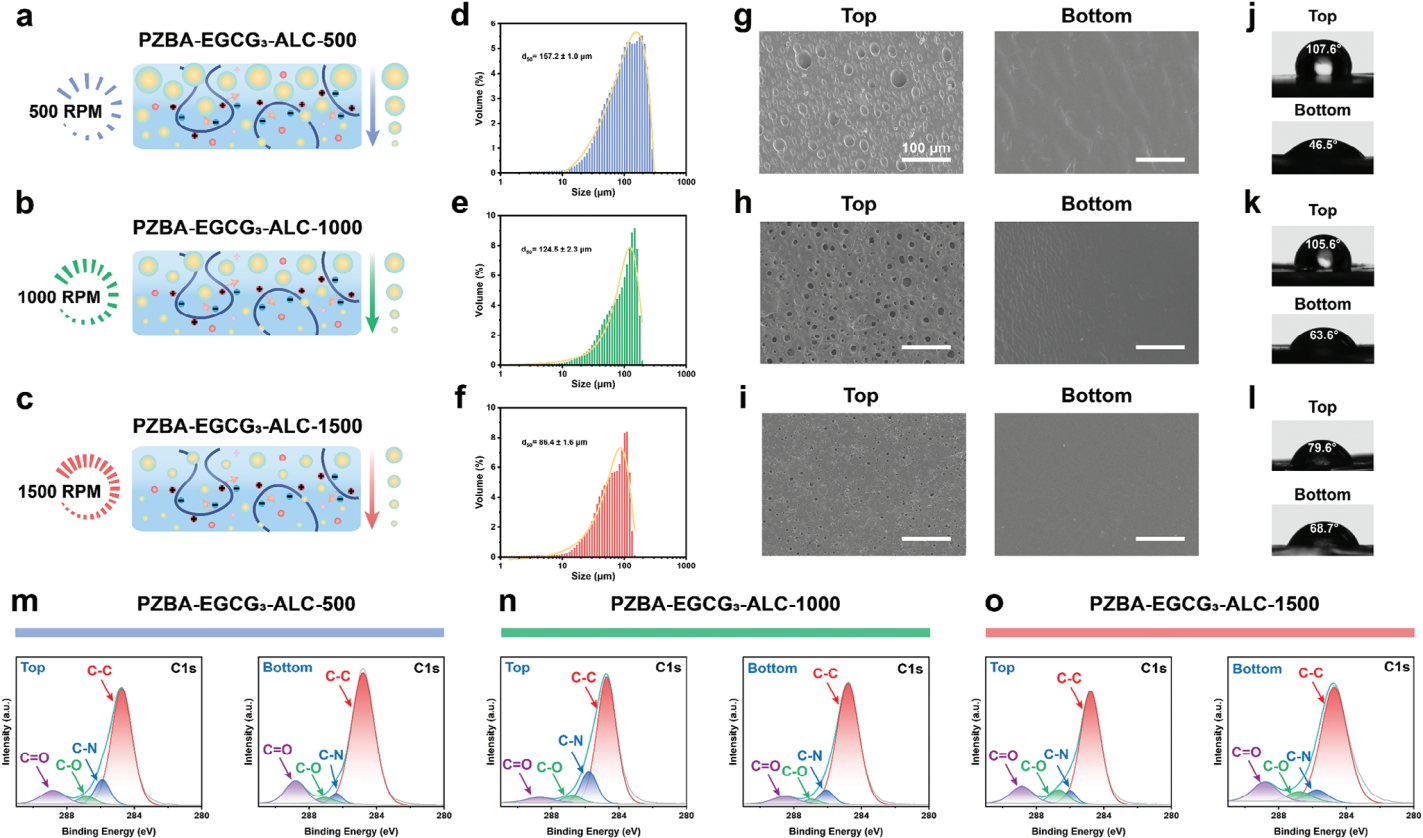
Characterization of asymmetric mechanism for PZBA‐EGCG‐ALC Janus hydrogels. a–c) Schematic illustration of the asymmetric structure for PZBA‐EGCG‐ALC Janus hydrogel precursor emulsions at different stirring speeds from 500 to 1500 RPM. d–f) The particle size characterization of the hydrogel precursor emulsions at different stirring speeds from 500 to 1500 RPM. g–i) The SEM pictures of the top and bottom surface morphology for the hydrogels at different stirring speeds from 500 to 1500 RPM. j–l) The water contact angle results for the hydrogels at different stirring speeds from 500 to 1500 RPM. m–o) Peak‐fitting XPS spectra in the C1s regions of the top surface and bottom surface for the hydrogels from 500 to 1500 RPM.

Through regulating the stirring speeds of the hydrogel precursor emulsions, different gradient‐sized LMA@CTAB emulsion droplets could be generated, thereby obtaining a series of PZBA‐EGCG‐ALC Janus hydrogels (PZBA‐EGCG_3_‐ALC‐500, PZBA‐EGCG_3_‐ALC‐1000, PZBA‐EGCG_3_‐ALC‐1500) with varying degrees of asymmetric properties (**Figure** **1**a–c). The LMA@CTAB emulsion droplet sizes of different hydrogel precursor emulsions were measured by the laser particle sizer (Figure 1d–f). With increasing stirring speed from 500 RPM to 1500 RPM, the LMA@CTAB emulsion droplet sizes of different hydrogel precursor emulsions gradually decreased. The average emulsion droplet sizes (d_50_) of the precursor emulsions of PZBA‐EGCG_3_‐ALC‐500, PZBA‐EGCG_3_‐ALC‐1000, and PZBA‐EGCG_3_‐ALC‐1500 hydrogels were 157.2 ± 1.0 µm, 124.5 ± 2.3 µm, and 86.4 ± 1.6 µm, respectively, which demonstrated that the stirring speeds could effectively regulate the formation of different gradient‐sized LMA@CTAB emulsion droplets. And the precursor emulsions remained stable within 24 h without a large change in their droplet sizes (Figure S4, Supporting Information). Since the density of LMA@CTAB emulsion droplets is smaller than that of water, larger sized LMA@CTAB emulsion droplets are more likely to float on the surface of the precursor emulsions. 3D optical images (Figure S5, Supporting Information) showed that the size of LMA@CTAB emulsion droplets on the top surface of PZBA‐EGCG‐ALC Janus hydrogels was all larger than that of the bottom surface. Subsequently, under UV polymerization, the gradient‐sized LMA@CTAB emulsion droplets could also be used as cross‐linking sites to form Janus hydrogels. The top and bottom surface morphology of PZBA‐EGCG‐ALC Janus hydrogels was observed by scanning electron microscopy (SEM) (Figure 1g–i). With increasing stirring speed from 500 RPM to 1500 RPM, the porous structure on the top surface of the PZBA‐EGCG‐ALC Janus hydrogels gradually shrunk, while the bottom surfaces were relatively flat. Figure S6 (Supporting Information) showed a representative section morphology of the PZBA‐EGCG‐ALC Janus hydrogel, which exhibited a distinctive asymmetric microstructure. In addition to the asymmetric surface morphology, we also tested the hydrophilicity and hydrophobicity of the top and bottom surfaces of the PZBA‐EGCG‐ALC Janus hydrogels (Figure 1j–l). All the PZBA‐EGCG‐ALC Janus hydrogels had larger contact angles on their top surfaces compared to their bottom surfaces. With increasing stirring speed from 500 RPM to 1500 RPM, the water contact angle on the top surface of the hydrogel decreased from 107.6° (PZBA‐EGCG_3_‐ALC‐500) to 79.6° (PZBA‐EGCG_3_‐ALC‐1500), and that on the bottom surface increased from 46.5° (PZBA‐EGCG_3_‐ALC‐500) to 68.7° (PZBA‐EGCG_3_‐ALC‐1500). The chemical compositions of the top and bottom surfaces of the hydrogels were also measured by using an X‐ray photoelectron spectroscopy (XPS). With increasing stirring speed from 500 RPM to 1500 RPM, on the bottom side of each PZBA‐EGCG‐ALC Janus hydrogel, we observed an increase in C = O intensity (288.9 eV) and a decrease in C‐N intensity (285.7 eV) (Figure 1m–o).^[^ ^30^ ^]^ Meanwhile, the intensity difference between ‐N_quat_R_3_ (401.8 eV) and ‐NR_2_ (399.6 eV) on the top surface of the PZBA‐EGCG‐ALC Janus hydrogels was higher than that on the bottom surface (Figure S7, Supporting Information).^[^ ^38^ ^]^ The above results indicated that compared to the bottom surface of the PZBA‐EGCG‐ALC Janus hydrogels, there were a lot of large‐sized hydrophobic LMA@CTAB emulsion droplets on their top surface. Through the gradient barrier effect of LMA@CTAB emulsion droplets, the ‐COOH groups in PAAc and cationic‐π structures in PZBA‐ECCG prodrug macromolecular networks were exposed more to the bottom surface of the PZBA‐EGCG‐ALC Janus hydrogels. With increasing stirring speed from 500 to 1500 RPM, the droplet sizes of LMA@CTAB emulsion droplets became smaller and the gradient size distributions became narrower, resulting in a reduced difference between the top and bottom surfaces of the PZBA‐EGCG‐ALC Janus hydrogels. Therefore, the interfacial distribution of free ‐COOH groups and cationic‐π structures on both sides of the PZBA‐EGCG‐ALC Janus hydrogels could be effectively controlled by regulating the stirring speed of the hydrogel precursor emulsions, thus obtaining hydrogels with different asymmetric behaviors.

### 2.3 Mechanical Properties of PZBA‐EGCG‐ALC Janus Hydrogels

**Figure 2:**
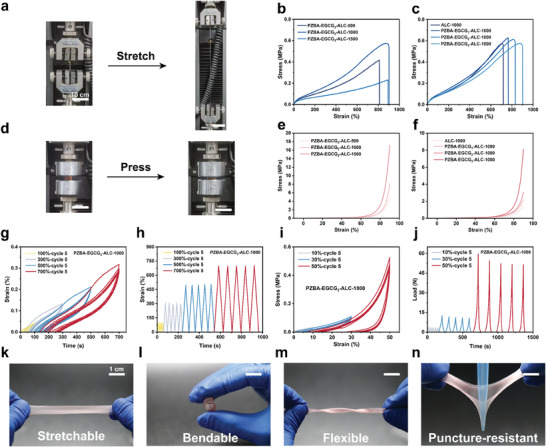
Characterization of mechanical properties for different Janus hydrogels. a) Digital photographs of tensile stress‐strain experiments and b) the stress‐strain curves for different Janus hydrogels at different stirring speeds from 500 to 1500 RPM, or c) varying different EGCG amounts at 1000 RPM. (*n*  =  3 independent samples) d) Digital photographs of compressive stress‐strain experiments and e) the curves for different Janus hydrogels at different stirring speeds from 500 to 1500 RPM, or f) varying different EGCG amounts at 1000 RPM. (*n*  =  3 independent samples) g) Successive cyclic tensile stress‐strain curves of the PZBA‐EGCG_3_‐ALC‐1000 hydrogel. (*n*  =  3 independent samples) h) Successive cyclic tensile test parameters of the PZBA‐EGCG_3_‐ALC‐1000 hydrogel fixed at different strains. i) Successive cyclic compressive stress‐strain curves of the PZBA‐EGCG_3_‐ALC‐1000 hydrogel. (*n*  =  3 independent samples) j) Successive cyclic compressive test parameters of the PZBA‐EGCG_3_‐ALC‐1000 hydrogel fixed at different strains. k‐n) Digital photographs of mechanical properties of the PZBA‐EGCG_3_‐ALC‐1000 hydrogel. The PZBA‐EGCG_3_‐ALC‐1000 hydrogel was stretchable, bendable, flexible, and puncture‐resistant.

Ruptured tendons are limited in their ability to resist tension and absorb shock.^[^ ^1^ ^]^ Since ruptured tendons are subjected to transient and continuous cyclic mechanical stimulation during daily use, it is of great importance to match the dynamic mechanics of hydrogels with the rupture mechanics of tendons and to maintain their structural and mechanical stability during long‐term implantation.^[^ ^39^ ^]^ For this purpose, we conducted tensile and compressive experiments to confirm the mechanical properties of PZBA‐EGCG‐ALC Janus hydrogels (**Figure** **2**a,d). With increasing stirring speed from 500 RPM to 1500 RPM, the elongation at break of the hydrogels gradually increased while the tensile strength first increased and then decreased (Figure 2b; Table S2, Supporting Information). Meanwhile, the compressive stress of the hydrogels gradually increased at the same deformation (Figure 2e; Table S4, Supporting Information). As mentioned before, at higher stirring speed, LMA@CTAB emulsion droplets were obtained with smaller particle size and narrower gradient size distribution. Moreover, LMA@CTAB emulsion droplets also act as cross‐linking sites in the subsequently formed PAAc network. Within a certain stirring speed range (i.e., 500 RPM–1000 RPM), increasing the cross‐linking density of the hydrogels could improve their mechanical properties, while excessive cross‐linking density (i.e., 1500 RPM) could instead restrict molecular chain movement in the hydrogel networks, leading to a decrease in their mechanical properties. In addition, the amount of EGCG in PZBA‐EGCG prodrug macromolecular networks also affected the mechanical properties of the hydrogels. With gradually increasing EGCG amount in the hydrogel networks at 1000 RPM, the tensile and compressive results show a similar trend to increasing stirring speeds (Figure 2c,f; Tables S3 and S5, Supporting Information). Compared with ALC‐1000 Janus hydrogel, the mechanical properties of PZBA‐EGCG‐ALC Janus hydrogels were significantly enhanced. There might be electrostatic interactions between the positively charged PZBA‐EGCG prodrug macromolecular networks and the negatively charged PAAc networks. Simultaneously, the EGCG amount would affect the formation of dynamic borate bonds in PZBA‐EGCG prodrug macromolecular networks. More dynamic bonds can effectively dissipate more external stresses, resulting in better mechanical properties of the hydrogels. With appropriate tensile and compressive strengths, the PZBA‐EGCG_3_‐ALC‐1000 hydrogel was selected for successive cyclic tensile and compressive testing (Figure 2g–j). After applying successive tensile or compressive strains, the PZBA‐EGCG_3_‐ALC‐1000 hydrogel could recover to its original state. The obvious hysteresis loop indicated that the hydrogels had good energy dissipation capabilities. To visualize the mechanical properties of the PZBA‐EGCG‐ALC Janus hydrogel, as shown in Figure 2k–n, the hydrogels could withstand large tensile, bending, twisting, and puncturing without their structure being damaged. In addition, the swelling properties of hydrogels are also important for internal wound applications in wet tissue environments. We tested the equilibrium swelling properties of PZBA‐EGCG‐ALC Janus hydrogels in PBS solution at 37 °C (Figure S8, Supporting Information). Compared to the ALC‐1000 Janus hydrogel without the PZBA‐EGCG prodrug macromolecular networks (354.4 ± 16.3%), all PZBA‐EGCG‐ALC Janus hydrogels exhibited limited swelling inhibition effect (e.g., 267.2 ± 15.8% for the PZBA‐EGCG_3_‐ALC‐1000 hydrogel). And the inhibition effect became more and more obvious with gradually increasing EGCG amount in the hydrogel networks at 1000 RPM. This might be attributed to the formation of more electrostatic interactions between the positively charged PZBA‐EGCG prodrug macromolecular networks and the negatively charged PAAc networks. Overall, the obtained PZBA‐EGCG‐ALC Janus hydrogels exhibited excellent mechanical compliance and efficient energy dissipation capability, which could withstand external tension and compression without implantation failure in humid and dynamic physiological environments.

### 2.4 Asymmetric Adhesion Characterization of the PZBA‐EGCG‐ALC Janus Hydrogels

**Figure 3:**
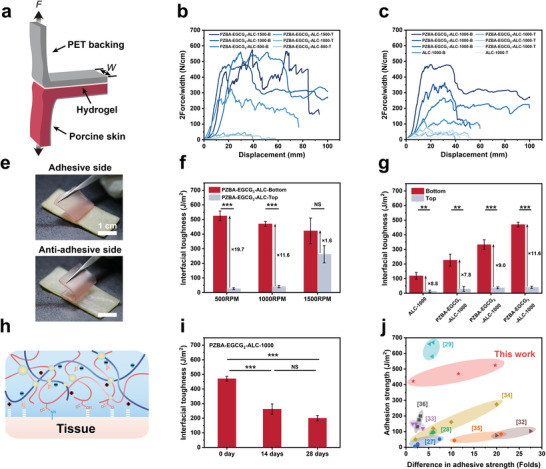
Characterization of asymmetric adhesion for different Janus hydrogels. a) Schematic illustration of 180° peeling test for different Janus hydrogels. b) 180° peeling test curves for different Janus hydrogels on wet porcine skin at different stirring speeds from 500 to 1500 RPM, or c) varying different EGCG amounts at 1000 RPM. e) Digital photographs showing the distinctly asymmetric adhesion performances to wet porcine skin using different sides of the PZBA‐EGCG_3_‐ALC‐1000 hydrogel. f) Interfacial toughness for different Janus hydrogels on porcine skin at different stirring speeds from 500 to 1500 RPM, or g) varying different EGCG amounts at 1000 RPM. h) Schematic illustration of the hydrogel adhesion mechanism, which includes synergetic effects of hydrogen bonds, electrostatic interactions, and covalent bonds. i) Interfacial toughness of the PZBA‐EGCG_3_‐ALC‐1000 hydrogel to porcine skin at different storage time. j) Comparison of adhesion strength and difference in adhesive strength for the PZBA‐EGCG‐ALC Janus hydrogels with previously reported Janus hydrogels. Values and error bars in (b, c, f, g, and i) represent the mean and standard deviation (*n*  =  5 independent samples). Statistical significance and *p* values were determined using a two‐tailed Student's *t*‐test with unequal variance: NS *p* > 0.05; ^*^ *p* ≤ 0.05; ^**^ *p* ≤ 0.01; ^***^ *p* ≤ 0.001.

According to the previous discussion on the asymmetric mechanism, through the gradient barrier effect of LMA@CTAB emulsion droplets, the ‐COOH groups in PAAc and cationic‐π structures in PZBA‐ECCG prodrug macromolecular networks were exposed more to the bottom surface of the PZBA‐EGCG‐ALC Janus hydrogels, which could achieve robust wet adhesion to tissues through the synergetic effects of hydrogen bonds, electrostatic interactions and covalent bonds. To this end, to simulate the adhesion state under a realistic internal physiological environment, we evaluated the adhesion performances of the top and bottom surfaces of the PZBA‐EGCG‐ALC Janus hydrogels on porcine skins via a 180° peel test (**Figure** **3**a). As shown in Figure 3b–g, the adhesion strength of the bottom surface of all hydrogels was higher than that of the top surface. Moreover, we found that the stirring speed had a significant effect on the asymmetric adhesion performances (Figure 3b,f). With increasing stirring speed from 500 RPM to 1500 RPM, the adhesion strength of the bottom surfaces of the hydrogels gradually decreased (from 524.8 ± 33.1 J m^−2^ to 422.3 ± 88.3 J m^−2^), the adhesion strength of their top surfaces gradually increased (from 26.7 ± 6.2 J m^−2^ to 261.7 ± 58.7 J m^−2^), which resulted in a gradual decrease in the difference in adhesion strength between the two surfaces (from 19.7‐fold to 1.6‐fold). In addition, the amount of EGCG in PZBA‐EGCG prodrug macromolecular networks also affected the adhesion strength of the hydrogels (Figure 3c,g). With gradually increasing EGCG amount in the hydrogel networks at 1000 RPM, the adhesion strength of the bottom and top surfaces of the hydrogels both gradually increased (from 119.0 ± 23.0 J m^−2^ to 470.1 ± 16.2 J m^−2^ for the bottom surfaces and from 13.5 ± 7.5 J m^−2^ to 40.5 ± 7.4 J m^−2^ for the top surfaces). However, the difference in adhesion strength between the top and bottom surfaces of the hydrogels was little changed (nearly tenfold or so). For daily storage scenarios at room temperature, the adhesion interface stability of the hydrogels is equally important. Although the adhesion strength of the PZBA‐EGCG_3_‐ALC‐1000 hydrogel gradually decreased as its storage time increased, it could still maintain a relatively robust adhesion strength after 28 days (200.0 ± 18.3 J m^−2^). Significantly, compared with existing Janus hydrogels, the obtained PZBA‐EGCG‐ALC Janus hydrogels combined a higher adhesion strength (up to 524.8 ± 33.1 J m^−2^) with a higher difference in adhesion strength (nearly 20‐fold) (Figure 3j).

Based on the above results, we hypothesized the asymmetric adhesion mechanism of hydrogels as follows. Since the density of LMA@CTAB emulsion droplets is smaller than that of water, larger sized LMA@CTAB emulsion droplets are more likely to float on the surface of the precursor emulsions. Through the gradient barrier effect of LMA@CTAB emulsion droplets, the ‐COOH groups in PAAc and cationic‐π structures in PZBA‐ECCG prodrug macromolecular networks were exposed more to the bottom surface of the PZBA‐EGCG‐ALC Janus hydrogels, which could achieve robust wet adhesion to tissues through the synergetic effects of hydrogen bonds, electrostatic interactions and covalent bonds (Figure 3h). With increasing stirring speed from 500 to 1500 RPM, the droplet sizes of LMA@CTAB emulsion droplets became smaller and the gradient size distributions became narrower, resulting in a reduced difference in adhesion strength between the top and bottom surfaces of the PZBA‐EGCG‐ALC Janus hydrogels. Catechol‐rich EGCG is well known to form a variety of non‐covalent interactions and covalent bonds with ‐NH_2_ and ‐SH on the surface of tissues.^[^ ^40^ ^]^ With gradually increasing EGCG amount in the hydrogel networks at 1000 RPM, the cationic‐π structures in PZBA‐ECCG prodrug macromolecular networks gradually increased, leading to an increase in adhesion strength of the top and bottom surfaces of PZBA‐ECCG‐ALC Janus hydrogels. Overall, the PZBA‐EGCG‐ALC Janus hydrogels could achieve the difference in adhesion strength between the two sides from 1.6 to 19.7 folds while maintaining a strong adhesion strength on their bottom sides (> 400 J m^−2^). This one‐step formed PZBA‐EGCG‐ALC Janus hydrogels provided a more convenient suture‐free strategy for promoting high‐quality tendon healing and preventing postoperative adhesions. Meanwhile, combining the results of mechanical and asymmetric adhesion properties, we concluded that the PZBA‐EGCG‐ALC Janus hydrogels obtained at 1000 RPM possessed suitable mechanical strength, adhesion strength, and difference in adhesion strength, which were used as subsequent evaluations in cell and animal experiments.

### 2.5 In Vitro ROS and RNS Scavenging Ability, Biocompatibility, and In Vivo Hemostatic Properties of the PZBA‐EGCG‐ALC Janus Hydrogels

**Figure 4:**
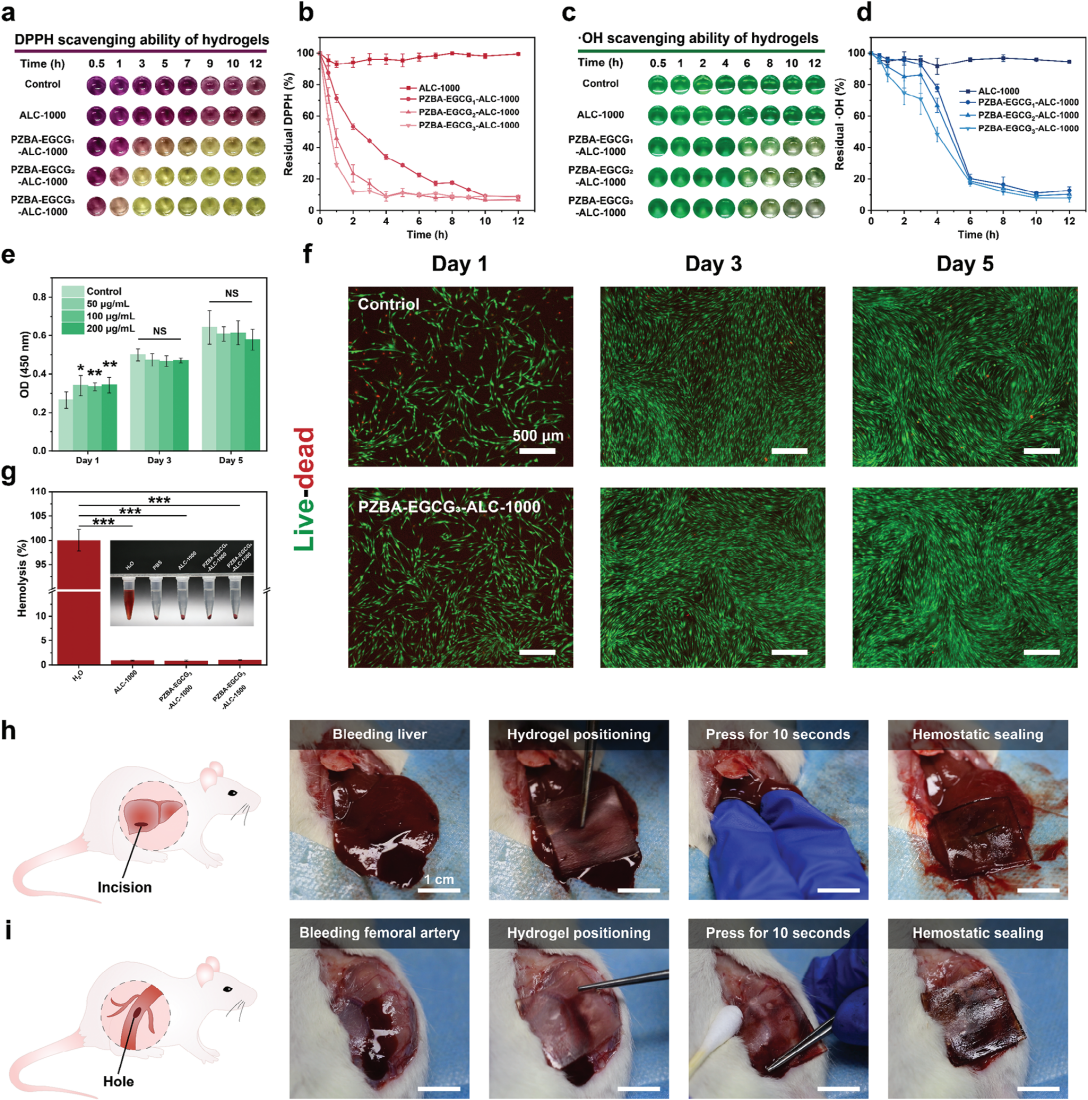
Characterization of in vitro ROS and RNS scavenging ability, biocompatibility, and in vivo hemostatic properties for different Janus hydrogels. a, c) Digital photographs and b, d) scavenging abilities of DPPH (0.1 mM) and ·OH (1 mM) radicals for different Janus hydrogels. e) Cell viability of the HDFs after incubation with the PZBA‐EGCG_3_‐ALC‐1000 hydrogel extracts for 24 h, 72 h, and 120 h. f) Representative live/dead staining images of HDFs after incubation with the PZBA‐EGCG_3_‐ALC‐1000 hydrogel extracts (200 µg mL^−1^) for 24 h, 72 h, and 120 h. g) Hemolysis rates and digital photographs (inset pictures) of different hydrogels after coincubation of different Janus hydrogels extract with 2% blood for 4 h. h) Photographs of the in vivo application of the PZBA‐EGCG_3_‐ALC‐1000 hydrogel to repair an actively bleeding defect in the liver of a rat. i) Photographs of the in vivo application of the PZBA‐EGCG_3_‐ALC‐1000 hydrogel to repair an actively bleeding defect in the femoral artery of a rat. For each animal model presented in h‐i, two independent experiments were conducted with similar results. Values and error bars in (a‐g) represent the mean and standard deviation (*n*  =  3 independent samples). Statistical significance and *p* values were determined using a two‐tailed Student's *t*‐test with unequal variance: NS *p* > 0.05; ^*^ *p* ≤ 0.05; ^**^ *p* ≤ 0.01; ^***^ *p* ≤ 0.001.

After Achilles tendon surgery, excessive ROS at the injured site during the first 24 h could generate a high level of oxidative stress, which might induce apoptosis and trigger an inflammatory response, resulting in severe clinical complications and a decrease in the quality of tendon healing. To reverse this trend, the PZBA‐EGCG prodrug macromolecules were introduced to offer more electrostatic interactions and ROS response capabilities for the formed hydrogel networks. Hydroxyl radical (·OH), and 2,2‐Diphenyl‐1‐(2,4,6‐trinitrophenyl)‐hydrazyl (DPPH) were used to determine the ROS and RNS scavenging abilities of the PZBA‐EGCG‐ALC Janus hydrogels (**Figure** **4**a–d). As shown in Figure 4a,b, ≈91.2 ± 2.0% (PZBA‐EGCG_3_‐ALC‐1000) and 90.9 ± 3.1% (PZBA‐EGCG_2_‐ALC‐1000) of DPPH were consumed within the first 4 h, which was about 1.4 folds faster than that of PZBA‐EGCG_1_‐ALC‐1000 hydrogel (65.9 ± 1.6%). The PZBA‐EGCG‐ALC Janus hydrogels scavenged almost all DPPH within 12 h, while the ALC‐1000 Janus hydrogel had little scavenging ability. As shown in Figure 4c,d, a similar trend was observed for the ·OH scavenging ability. The PZBA‐EGCG‐ALC Janus hydrogels also scavenged almost all ·OH within 12 h. Furthermore, with gradually increasing EGCG amount in the hydrogel networks at 1000 RPM, the ·OH scavenging rate of the hydrogels was gradually accelerated at the same time point. The above results suggested the presence of ROS‐responsive PZBA‐EGCG prodrug macromolecules in the PZBA‐EGCG‐ALC Janus hydrogel networks, which could be used as effective ROS scavengers and inhibit their accumulation around injured Achilles tendons, alleviate oxidative damage and promote subsequent tendon reconstruction.

In addition to suitable antioxidant and anti‐inflammatory properties, in vivo implantable biomaterials for tissue repair must have excellent biocompatibility and degradability. Since human dermal fibroblasts (HDFs) are derived from the same mesoderm as tendon cells. They have no significant differences in cell morphology and secrete similar cell matrix components.^[^ ^8^, ^41^ ^]^ Moreover, fibroblasts are widely available and proliferate rapidly, thus we used HDFs to evaluate the biocompatibility of the hydrogel with the tendon tissue over time. Initially, the proliferation of HDFs was assessed with CCK‐8 assay, which revealed a normal proliferation rate in each group, with no significant difference on the 5th day (Figure 4e). Then, live/dead cell staining was performed on HDFs cultured for 1, 3, and 5 days. As shown in Figure 4f, HDFs in both groups had good cell morphology. Although a small number of dead cells appeared, the overall proliferation rate was within the expected range. Meanwhile, the observed differences did not reach statistical significance. Furthermore, we evaluated the in vitro hemocompatibility of the hydrogels. As shown in Figure 4g, the hemolysis rates of all hydrogels were less than 5% relative to the positive control group (H_2_O). To evaluate the in vivo degradability of the hydrogels, PZBA‐EGCG_3_‐ALC‐1000 Janus hydrogels were implanted subcutaneously on the dorsal regions of mice. As shown in Figure S9 (Supporting Information), the volumes of hydrogels were gradually decreasing over time, with ≈76% degradation on the 21th day. According to the above results, the PZBA‐EGCG‐ALC Janus hydrogel exhibited excellent biocompatibility and degradability. It could be retained in vivo for at least 21 days, which would promote tendon healing while providing an effective anti‐adhesion barrier.

Severe hemorrhages may occur owing to operational errors during surgery. Their management is a particularly difficult challenge due to the complexity and time‐sensitivity of bleeding injuries. To demonstrate the versatility of the PZBA‐EGCG‐ALC Janus hydrogels, we conducted a series of in vivo hemostasis experiments by applying the PZBA‐EGCG_3_‐ALC‐1000 hydrogel to liver and femoral artery defects in live rats (Figure 4h,i). After 10 s of gentle pressing, the PZBA‐EGCG_3_‐ALC‐1000 hydrogel achieved blood‐resistant sealing of the bleeding liver injury (a ≈5 mm‐long, ≈2 mm‐deep incision) and the femoral artery injury (a ≈2 mm‐diameter hole) (Movies S1 and S2, Supporting Information). In conclusion, the PZBA‐EGCG‐ALC Janus hydrogels demonstrated an additional potential for the treatment of intraoperative bleeding.

### 2.6 In Vivo Study of the PZBA‐EGCG‐ALC Janus Hydrogels for Suture‐Free and High‐Quality Tendon Healing

**Figure 5:**
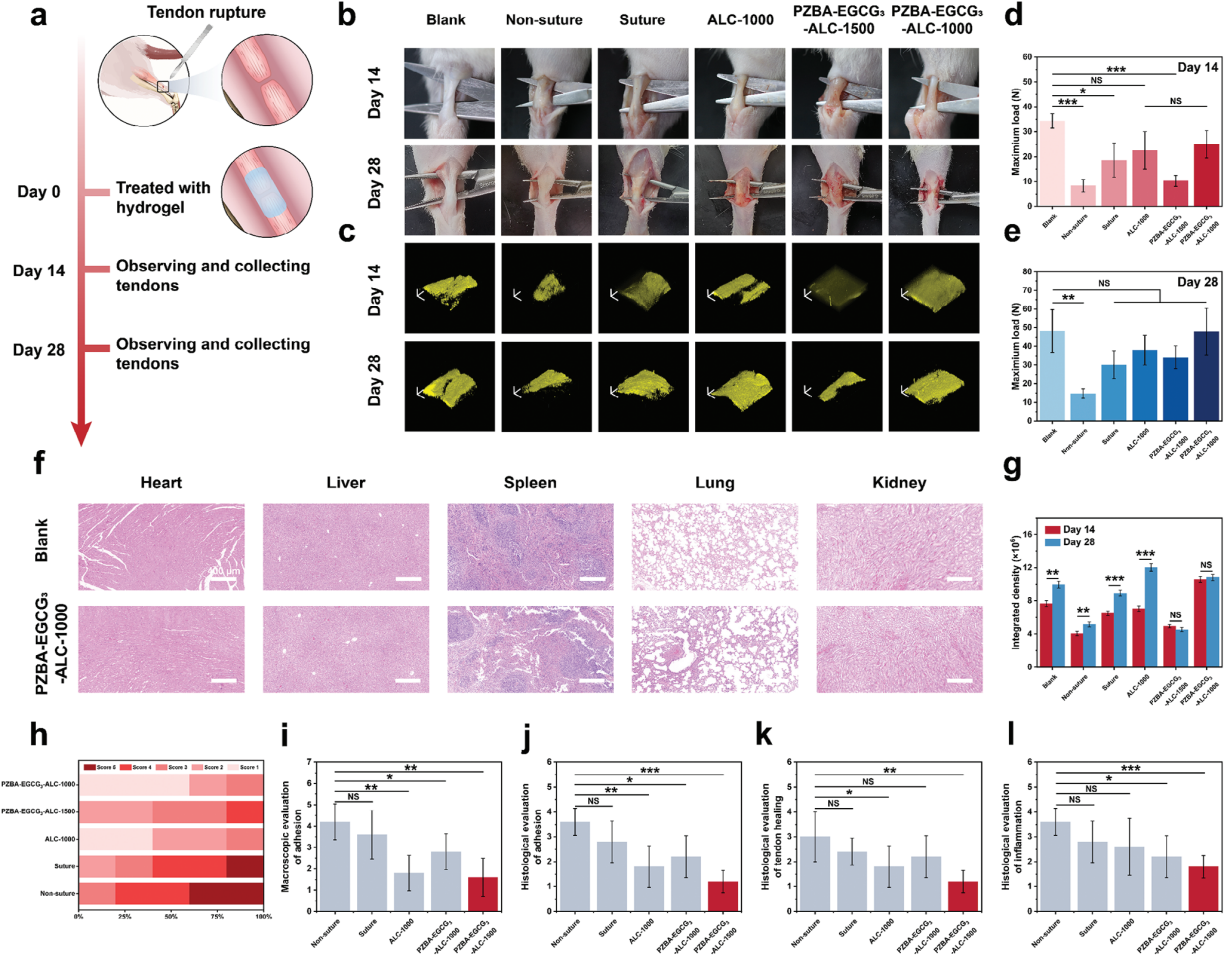
In vivo study of the PZBA‐EGCG‐ALC Janus hydrogels for suture‐free and high‐quality tendon healing. a) Schematic illustration of the rat Achilles tendon rupture model establishment and prevention of postoperative peritendinous adhesions by PZBA‐EGCG‐ALC Janus hydrogels. b) Gross observation of peritendinous adhesion of Achilles tendon rupture after 14 days and 28 days. c) Representative two‐photon microscopy 3D reconstructed images of the healed tendons on the 14th and 28th day. Collagen fiber second harmonic generation (SHG) is shown in yellow, and 3D reconstructions of the elastic fiber network from the full image stack. Scale bar, 500 µm. Maximum rupture force of the healed Achilles tendon tissues on the d) 14th and e) 28th day. f) H&E staining images of the major organs from SD rats after treatment with the PZBA‐EGCG_3_‐ALC‐1000 hydrogels for 28 days. g) Integrated fluorescence intensities of two‐photon microscopy 3D reconstructed images of the healed tendons on the 14th and 28th day. h) Distribution of adhesion scores and i) adhesion assessment based on the gross observation on the 28th day. Histological evaluation of j) peritendinous adhesion, k) tendon healing quality, and l) inflammation reaction based on the H&E‐stained images on the 28th day. For each animal model presented in a‐c, five independent experiments were conducted with similar results. Values and error bars in (d‐l) represent the mean and standard deviation (*n*  =  5 independent samples). Statistical significance and *p* values were determined using a two‐tailed Student's *t*‐test with unequal variance: NS *p* > 0.05; ^*^ *p* ≤ 0.05; ^**^ *p* ≤ 0.01; ^***^ *p* ≤ 0.001.

To evaluate the practical therapeutic efficacy of the developed PZBA‐EGCG‐ALC Janus hydrogels, we established a rat Achilles tendon rupture model and then wrapped the tendons with hydrogels (**Figure** **5**a; Figure S10, Supporting Information). To evaluate the effects of time dimensional factors (early inflammatory response around the injury sites) as well as spatial dimensional factors (mechanical compliance and asymmetric adhesion behavior) on tendon healing, we selected **ALC‐1000** (Janus hydrogel without PZBA‐EGCG prodrug macromolecules), **PZBA‐EGCG_3_‐ALC‐1500** (non‐Janus hydrogel with PZBA‐EGCG prodrug macromolecules), and **PZBA‐EGCG_3_‐ALC‐1000** (Janus hydrogel with PZBA‐EGCG prodrug macromolecules) hydrogels as experimental groups. Meanwhile, **non‐suture** (partially‐ruptured tendons without any treatment) and **suture** (partially‐ruptured tendons repaired with suture) groups were performed as two control groups to investigate the efficacy of our suture‐free strategy. As shown in Figure 5b, the skin conditions of the local healing sites were carefully examined on the 14th and 28th postoperative days, and no serious ulcers or infections around the wounds were found in any of the groups. Furthermore, after treatment with the PZBA‐EGCG‐ALC‐1000 Janus hydrogels for 28 days, there were no significant inflammation or lesions in the major organs of the rats (i.e., heart, liver, spleen, lungs, and kidneys), which were similar to blank group (Figure 5f). For the non‐suture group, a large amount of fibrous tissues formed around the healed tendon, requiring manual separation of the tendon and its surrounding tissues with a scalpel. Compared to the non‐suture group, in the suture and PZBA‐EGCG_3_‐ALC‐1500 groups, although there were less adhesions formed, the tendon surfaces were still covered by bundles of fibrous tissues tightly interwoven with the subcutaneous tissues. In contrast, for the ALC‐1000 and PZBA‐EGCG_3_‐ALC‐1000 groups, there were few dense adhesions formed in the peritendinous area. In addition, through 3D reconstructed images of the healed tendons by two‐photon microscopy and their integrated fluorescence intensity analysis, we observed differences in the arrangement and content of collagen in each group. As shown in Figure 5c,g, compared to the 14th day, the fluorescence intensity of the healed tendons in each group was higher than that on the 28th day, indicating an increase in collagen content. On postoperative 28th day, limited or disordered arrangement of collagen was observed in the non‐suture, suture, and PZBA‐EGCG_3_‐ALC‐1500 groups. Since fibrous scar tissues are not visualized under two‐photon laser excitation, we hypothesized that there were more scar tissues in those healed tendons. For the ALC‐1000 and PZBA‐EGCG_3_‐ALC‐1000 groups, the healed tendons exhibited relatively strong and ordered fluorescence, especially in the PZBA‐EGCG_3_‐ALC‐1000 group, which was comparable to that of the blank group. Tendon adhesion was quantitatively analyzed using a scoring system based on gross observations as well as two‐photon microscopy image results. The results showed that the adhesion score of the PZBA‐EGCG_3_‐ALC‐1000 Janus hydrogel was the lowest among all groups (1.60 ± 0.89), further confirming its more optimal suture‐free strategy for promoting high‐quality tendon healing and preventing postoperative adhesions (Figure 5h,i). The effect of PZBA‐EGCG‐ALC Janus hydrogels on the tendon healing process was monitored by evaluating their biomechanical properties. Consistent with the results of the gross observation evaluation and two‐photon microscopy images, on the 14th day, the tensile strength of the healed tendons in each group was lower than that of the blank group, indicating their insufficient collagen regeneration (Figure 5d; Figure S11, Supporting Information). On the 28th day, the tensile strength of PZBA‐EGCG_3_‐ALC‐1000 (47.91 ± 12.50 N) significantly exceeded that of the other groups (non‐suture: 14.74 ± 2.51 N; suture: 30.08 ± 7.50 N; ALC‐1000: 38.00 ± 8.02 N; PZBA‐EGCG_3_‐ALC‐1500: 34.15 ± 6.05 N), which was comparable to the blank group (48.22 ± 11.54 N), indicating its optimal recovery of the ruptured tendons (Figure 5e; Figure S12, Supporting Information).

### 2.7 Histology Assessment of Achilles Tendon Rupture Regeneration

**Figure 6:**
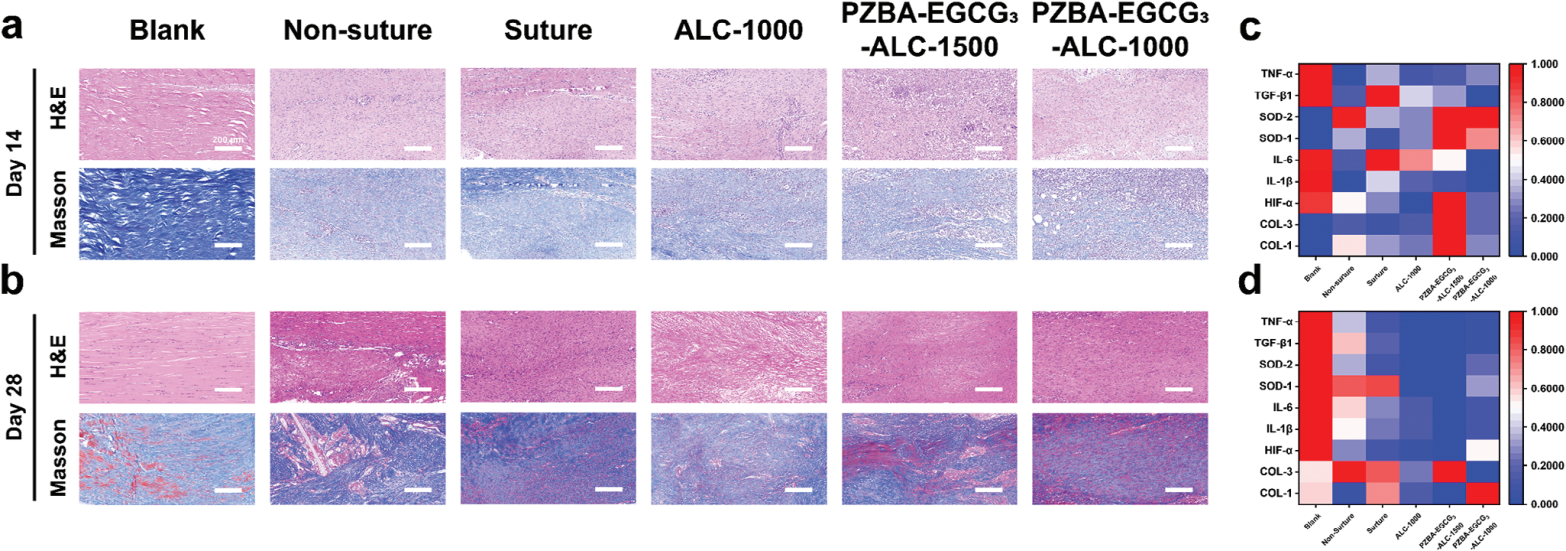
Histology assessment of Achilles tendon rupture regeneration. H&E and Masson staining of harvested tendon tissues on the a) 14th and b) 28th day after surgery. The heatmaps of COL‐1, COL‐3, TGF‐β1, IL‐1β, IL‐6, HIF‐1α, TNF‐α, SOD‐1, and SOD‐2 gene expressions of Achilles tendons on the c) 14th and d) 28th day.

Furthermore, the status of tendon healing in each group was also evaluated by histological staining with hematoxylin and eosin (H&E) and Masson's trichrome. As shown in **Figure** **6**a,b, compared to the 14th day, all groups except the blank and non‐suture groups on the 28th day exhibited reduced inflammatory cell infiltration and a denser and richer arrangement of collagen fibers. This demonstrated that the PZBA‐EGCG‐ALC Janus hydrogels could replace sutures to promote high‐quality tendon healing and preventing postoperative adhesions. However, the final healing efficacy was different. On the 28th day, H&E and Masson staining results showed that severe adhesion tissues formed in the non‐suture group, which entangled the healed tendon with granulation tissues and attracted a large amount of inflammatory cells, exhibiting a disordered collagen fiber arrangement (Figure 6b). For the suture and ALC‐1000 groups, some inflammatory cells appeared in the healed tendons. Only loose fiber bundles were formed in the peritendinous area, which could be due to the foreign body reaction induced by the sutures and the hydrogel. Notably, for the PZBA‐EGCG_3_‐ALC‐1500 group, there was reduced granulation tissue formation and decreased inflammatory cell infiltration, suggesting an in vivo anti‐inflammatory effect of the PZBA‐EGCG prodrug macromolecules. The PZBA‐EGCG_3_‐ALC‐1000 group showed the lowest fibrotic tissue formation and the fewest inflammatory cells, exhibiting a more regular and dense arrangement of collagen fibers and myofibers, which was comparable to the blank group. Further quantitative analysis of peritendinous adhesions, quality of tendon healing, and inflammatory responses based on the H&E and Masson staining results were shown in Figure 5j–l. We found that compared to the non‐suture group, the ALC‐1000 had lower peritendinous adhesion and tendon healing scores, whereas the PZBA‐EGCG_3_‐ALC‐1500 had lower tendon healing and inflammation scores. Notably, the PZBA‐EGCG_3_‐ALC‐1000 group had the lowest average scores on these three indexes (i.e., peritendinous adhesion score, tendon healing score, and inflammation score). This suggested that the PZBA‐EGCG_3_‐ALC‐1000 Janus hydrogel combined the benefits of ALC‐1000 (providing an effective mechanical support and physical barrier in the spatial dimension) and PZBA‐EGCG_3_‐ALC‐1500 (effectively minimizing early inflammation generation in the time dimension) hydrogels. As a result, it showed excellent anti‐adhesion and anti‐inflammatory effects in promoting tendon healing.

To further investigate the time‐space regulating mechanism of the hydrogels in promoting high‐quality tendon healing and preventing postoperative adhesions, tendon matrix components (COL‐1 and COL‐3), fibrosis markers (TGF‐β1), and inflammatory markers (IL‐1β, IL‐6, HIF‐1α, TNF‐α, SOD‐1, and SOD‐2) were tested by qRT‐PCR on the 14th (Figure 6c; Figure S13, Supporting Information) and 28th day (Figure 6d; Figure S14, Supporting Information). The gene expression results were transformed using the min‐max normalization method and scaled to the range of [0, 1]. The transformed results were then shown as heatmaps in Figure 6c,d (blue color represents relatively decreased concentration, whereas red color represents relatively increased concentration). Ruptured tendon sites are susceptible to invasion by fibroblasts and inflammatory cells.^[^ ^42^ ^]^ Inflammation and excessive fibrosis could lead to increased exogenous healing as well as adhesion formation.^[^ ^43^, ^44^ ^]^ During the early inflammation and proliferation phases (Figure 6c; Figure S13, Supporting Information), compared to the non‐suture, suture and ALC‐1000 groups, significant decreased levels of pro‐inflammatory and fibrosis factor expression (e.g., IL‐1β, IL‐6, HIF‐1α, TNF‐α, and TGF‐β1) and increased levels of anti‐inflammatory factor expression (e.g., SOD‐1 and SOD‐2) were observed in the PZBA‐EGCG_3_‐ALC‐1500 and PZBA‐EGCG_3_‐ALC‐1000 groups. On the 28th day, tendons in both the non‐suture and suture groups still maintained higher levels of oxidative stress and fibrosis (Figure 6d; Figure S14, Supporting Information). These inflammation and fibrosis analysis results were consistent with gross observations, two‐photon microscopy images, and histologic assessment results. They further demonstrated that the PZBA‐EGCG prodrug macromolecules of hydrogels could effectively alleviate the early inflammation in the time dimension, thus avoiding excessive exogenous healing of ruptured tendons while accelerating their transition from proliferation to remodeling phases.

In addition, COL‐1 accounts for nearly 60% weight of the native tendon matrix and plays a key role in bearing loads and restoring deformation.^[^ ^45^ ^]^ However, COL‐3 is massively secreted during the early inflammation and proliferation phases of tendon healing. If COL‐3 is overexpressed during the later remodeling phase, it will result in slender collagen fibers and impaired biomechanical strength.^[^ ^45^ ^]^ Figure 6d and Figure S14 (Supporting Information) showed that on the 28th day, both COL‐1 and COL‐3 expression in the healed tendons were higher in the suture group. Lower COL‐1 expression and higher COL‐3 expression in the healed tendons appeared in the non‐suture and PZBA‐EGCG_3_‐ALC‐1500 groups. However, this situation was reversed in the ALC‐1000 and PZBA‐EGCG_3_‐ALC‐1000 groups. These tendon matrix composition analysis results were consistent with gross observations, two‐photon microscopy images, and biomechanical results. They further demonstrated that the distinct asymmetric adhesion of hydrogels in the spatial dimension was the key factor in accelerating the gradual replacement process of COL III by COL I, thereby inhibiting exogenous healing while accelerating endogenous healing of ruptured tendons.

## 3 Conclusion

Taken together, by controlling the interfacial distribution of free ‐COOH groups and cationic‐π structures on both sides of the hydrogels, a series of PZBA‐EGCG‐ALC Janus hydrogels with varying degrees of asymmetric properties were successfully prepared using a simple and efficient one‐step synthesis method. It is worth noting that the Janus hydrogels could achieve a high difference in adhesion strength (nearly 20‐fold) while maintaining a strong adhesion strength on their bottom sides (up to 524.8 ± 33.1 J m^−2^). More importantly, with an efficient mechanical support and physical barrier in the spatial dimension and an effective minimization of early inflammation in the temporal dimension, the Janus hydrogels achieved high‐quality healing for ruptured tendons and successfully prevented postoperative adhesions within 28 days. The PZBA‐EGCG‐ALC Janus hydrogels with time‐space regulating properties offered a more convenient suture‐free strategy for high‐quality tendon healing. They are promising as a potential candidate material for life‐saving internal surgeries and show great potential for future clinical translation.

## 4 Experimental Section

### Materials

Hexachlorophosphazene (HCCP), AlCl_3_, *N*, *N*‐dimethyl‐1,2‐ethanediamine (DEMAEA), and triethylamine (TEA) were supplied by J&K Scientific Co., Ltd. (China). HCCP was purified by recrystallization from n‐hexane and subsequent vacuum sublimation at 55 °C. DEMAEA and TEA were dried over CaH_2_ and distilled before use. 3‐bromomethyl‐phenylboronic acid (BPBA), ethyl bromoacetate (EBA), acrylic acid (AAc), lauryl methacrylate (LMA), hexadecyl trimethyl ammonium bromide (CTAB), epigallocatechin gallate (EGCG), and lithium phenyl‐2,4,6‐trimethylbenzoylphosphinate (LAP) were purchased from Shanghai Aladdin Chemical Reagent Co., Ltd. (China) without purification. All other solvents and chemicals were purchased from commercial sources unless otherwise stated and used as received without any further purification. All porcine tissues were purchased from a local market. Tissue RNA Purification Kit PLUS, 4 × EZscript Reverse Transcription Mix II (with gDNA Remover), and 2 × EZ Color SYBR Green qPCR Master Mix were purchased from EZBioscience. Counting Kit‐8 (CCK‐8), and Live/Dead Cell Double Staining Kit were purchased from Solarbio.

### Animal

All animal experiments were performed according to the Guidelines for National Research Council's Guide for the Care and Use of Laboratory Animals. Male SD rats (200–220 g) and male C57BL/6 mice (19–21 g) were provided by the Experimental Animal Center of Zhejiang Academy of Medical Sciences, China. All animal protocols in this study were approved by the Animal Experimental Ethical Inspection of the First Affiliated Hospital, College of Medicine, Zhejiang University (Reference Number: 2022–676)

### Synthesis and Characterization of PZBA

The poly[(*N,N*‐dimethylethylenediamine)‐*g*‐(*N,N,N,N*‐dimethylaminoethyl‐*p*‐methylphenylboronic acid ammonium bromide‐*g*‐(*N,N,N,N*‐dimethylaminoethyl ethyl acetate ammonium bromide)] (PZBA) was synthesized according to our previous work.^[^ ^22^ ^]^ The synthesis route is shown in Figure S1 (Supporting Information). First, poly(dichlorophosphazene) (PDCP) was synthesized by thermal ring‐opening polymerization of purified HCCP under vacuum at 250 °C for 5 h. Then poly(*N*, *N*‐dimethyl‐1,2‐ethanediamine)phosphazene (PDAP) was obtained by DEMAEA nucleophilic substitution reaction in THF at 40 °C for 48 h (PDCP (unit): DMAEA = 1:3). After that, PZBA was subsequently obtained by a 2‐step nucleophilic substitution reaction to PDAP by BPBA and EBA in methanol at 40 °C for 48 h, respectively. (PDAP (unit): BPBA: EBA = 1:0.4:3). The mixture obtained from the above reactions was dialysis in deionized water for 5 days (MW = 3500). Finally, the purified PZBA was obtained by freeze‐drying. The chemical structures of PDAP, PPBA, and PZBA were confirmed by using proton and phosphorus nuclear magnetic resonance (^1^H and ^31^P NMR) spectroscopy (Bruker Ascend 600 MHz, Bruker Corporation, Switzerland) in deuterium oxide (D_2_O). Fourier transform infrared spectroscopy (FT‐IR) (Nicolet iS50, Thermo Scientific) were recorded in the range of 400–4000 cm^−1^ to further confirm the chemical structures of PZBA.

### Preparation and Characterization of the PZBA‐EGCG‐ALC Janus Hydrogels

*Preparation of the PZBA‐EGCG‐ALC Janus hydrogels*: Taking the PZBA‐EGCG_3_‐ALC‐1000 Janus hydrogel as an example, the surfactant CTAB (0.3749 g, 1.04 mmol) was completely dissolved in deionized water (5.0 mL) at 40 °C, and then AAc (4.4282 g, 61.06 mmol) and LMA (0.1763 g, 0.71 mmol) monomers, PZBA (0.2988 g, 0.60 mmol) and EGCG powder (0.0679 g, 0.15 mmol) were sequentially added to the above solutions and stirred for 4 h with a magnetic stirrer at 1000 RPM stirring speeds. Subsequently, LAP photoinitiator (0.0068 g, 0.02 mmol) was added and stirred continuously for 10 min to form hydrogel precursor emulsions. The resulting precursor emulsion was injected into a polytetrafluoroethylene mold (50 mm × 50 mm × 1 mm) and its upper surface was covered with a glass sheet. Finally, after placed for 1 h, it was crosslinked under 100 W UV light for 30 min. As a comparison, the ALC‐1000 Janus hydrogel was prepared in a similar way to the above steps without the addition of PZBA and EGCG powders. Each hydrogel was dialyzed in 1 L of deionized water for 5 days (changing the water twice a day) and then dried to the original weight in a 30 °C oven. The detailed preparation parameters of ALC‐1000, PZBA‐EGCG_3_‐ALC‐500, PZBA‐EGCG_3_‐ALC‐1000, PZBA‐EGCG_3_‐ALC‐1500, PZBA‐EGCG_1_‐ALC‐1000, and PZBA‐EGCG_2_‐ALC‐1000 Janus hydrogels is shown in Table S1 (Supporting Information).

*Particle Size Characterization of the Hydrogel Precursor Emulsions*: The size distribution of the hydrogel precursor emulsions was detected by the laser particle sizer (Coulter LS13320, Beckman) according to the wet dispersion technology.

*Morphologies of the Hydrogels*: Scanning electron microscopy (HITACHI SU‐3500 SEM) was used to observe the morphology of the hydrogels. Before observation, the hydrogels were frozen in liquid nitrogen and then lyophilized until the hydrogel qualities did not change. The dried hydrogels were sprayed with gold for 90 s before testing. The roughness of the top/bottom surfaces of the hydrogels was also measured by the 3D optical profilometer (GT‐K, Bruker).

*Characterization of the Chemical Compositions of the Hydrogels*: Fourier transform infrared (FT‐IR) spectra (Nicolet iS50, Thermo Scientific) were recorded in the range of 400–4000 cm^−1^ to further confirm the chemical compositions of the hydrogels. The chemical compositions of the top and bottom surfaces of the hydrogels were also measured by using an X‐ray photoelectron spectroscopy (XPS) (Thermo Scientific ESCALAB 250Xi, X‐ray energy 1486.6 eV, pass energy: 20 eV, step energy: 0.05 eV). The water contact angles of the top/bottom surfaces of the hydrogels were measured by the Video Contact Angle Tester (OCA 20, Dataphysics).

### Evaluation of Mechanical Performance

Tensile and compressive tests were performed by a mechanical testing machine (Instron‐2 kN). For tensile tests, the samples of dumbbell‐shaped strips (thickness 3 mm, width 2 mm, length 35 mm) were prepared. The tensile rate was fixed at 100 mm min^−1^ at room temperature. For compressive test, the shape of the hydrogel samples was cylindrical with 10 mm in diameter and 10 mm in height. And the compressive rate was fixed at 3 mm min^−1^. Successive cyclic tests were carried out by performing subsequent trials immediately after the initial loading. Load and displacement data were collected during the experiment. All the tests were repeated three times for each group.

### Evaluation of Swelling Behavior

To evaluate the swelling behavior of PZBA‐EGCG‐ALC Janus hydrogels, cylindrical hydrogels with 10 mm in diameter and 10 mm in height were immersed in 50 mL of phosphate‐buffered saline solution (PBS; pH = 7.4) at 37 °C. At each predetermined time point, the immersed samples were taken out and weighed. The swelling ratio was calculated by the following equation:

(1) $$\begin{array}{c} \mathrm{Swelling}\;\mathrm{ratio}\left(\%\right)=\frac{w_{t}-w_{0}}{w_{0}}\times 100\% \end{array}$$

where *w*_0_ is the weight of the original dry hydrogels, and *w_t_* is the weight of the swollen hydrogels at different time points. All the tests were repeated three times for each group.

### Evaluation of Tissue Adhesive Performances

Adhesion tests were performed on the inner surface of porcine skin washed with phosphate buffer solution (PBS). To measure interfacial toughness, tissue samples (width 20 mm, length 50 mm) were adhered to the various hydrogels (width 20 mm, length 30 mm, thickness 1 mm) and tested via the standard 180° peel test (ASTM F2256) using a mechanical testing machine (Instron‐2 kN). Polyethylene terephthalate (PET) films (width 20 mm, length 50 mm) were used as a stiff backing for the hydrogels. Adhered samples were kept in sealed bags to maintain a humid environment prior to measurement. All tests were conducted with a constant peeling speed of 50 mm min^−1^ and repeated five times for each group. The interfacial toughness was calculated according to the following equation:

(2) $$\begin{array}{c} \mathrm{Interfacial}\;\mathrm{toughness}=\frac{2\;\times \;F_{plateau}}{w}\; \end{array}$$

where *F_plateau_* is the plateau force during the peeling process, and *w* is the width of the adhesion area.

### Evaluation of In Vitro Reactive Oxygen Species (ROS) and Reactive Nitrogen Species (RNS) Scavenging Abilities

Hydroxyl radical (·OH), and 2,2‐Diphenyl‐1‐(2,4,6‐trinitrophenyl)‐hydrazyl (DPPH) were used to determine the ROS and RNS scavenging abilities.

*DPPH Scavenging Abilities*: The EtOH solution of DPPH (0.1 mM, 25 mL) was mixed with the PZBA‐EGCG‐ALC Janus hydrogels (50 mg). At fixed time points, the UV absorption intensity of the mixed solution at 517 nm was measured. All the tests were repeated three times for each group.

*·OH Scavenging Abilities*: The aqueous solution of ·OH (1 mM, 25 mL) was mixed with PZBA‐EGCG‐ALC Janus hydrogels (50 mg). At fixed time points, an equal amount of the mixture was mixed with DMSO solution of TMB (10  mM). Subsequently, the UV absorption intensity of the mixed solution at 904 nm was measured. All the tests were repeated three times for each group.

### Evaluation of In Vitro and In Vivo Biocompatibility

*Cytotoxicity Evaluation*: Cytotoxicity of the hydrogels was evaluated by co‐culturing with the human dermal fibroblasts (HDFs). Specifically, after 40 mg of hydrogel was extracted in 4 mL of DMEM complete medium for 24 h, 10 mg mL^−1^ extracts were obtained by filtration and sterilization using 0.22 µm filter membranes. The extracts were then diluted to 200, 100, and 50 µg ml^−1^. HDFs in logarithmic growth phase were taken and counted. The cell concentration was adjusted to 10^4^ per well and then seeded into 96‐well plates for further incubation in a thermostatic incubator (5% CO_2_, 37 °C) for 24 h, 72 h, and 120 h.

At each predetermined time point, 100 µL of each hydrogel extract replaced the medium in the 96‐well plate as the experimental group. The cell viability was quantified by using the CCK‐8 kit (Beijing Kulabo Technology Co., Ltd.). The absorbance was quantified using a microplate reader (Infinite 200 PRO, TECAN) at 450 nm. The live/dead staining method was also used to evaluate the cell viability by using a staining dye (Calcein‐AM/propidium iodide dye). All the tests were repeated three times for each group.

*Hemolysis Activity Assay*: The hemolysis activity of hydrogels was evaluated by the hemolysis ratio of anticoagulant bovine blood (Beijing Solarbio Science & Technology). The hydrogels were added to 500 µL of RBC suspension (5% PBS, v/v) at a final concentration of 1 mg mL^−1^. After incubation at 37 °C for 4 h, the samples were centrifuged at 2000 RPM for 10 min. H_2_O and PBS were used as positive and negative controls, respectively. The absorbance of the supernatants at 540 nm was measured by a microplate reader (ReadMax 1900) and the hemolysis rate was calculated according to the following equation:

(3) $$\begin{array}{c} \mathrm{Hemolysis}\;\mathrm{ratio}\;\left(\%\right)=\left(\frac{A_{s}-A_{0}}{A_{t}-A_{0}}\right) \end{array}\times 100\%$$

where *A_s_*, *A_0_*, and *A_t_* represent the absorbance of the sample, PBS, and H_2_O, respectively. All the tests were repeated three times for each group.

*In Vivo Degradability*: For in vivo degradation test, the PZBA‐EGCG‐ALC Janus hydrogel (5 × 5 × 0.5 mm^3^) was implanted subcutaneously on the dorsal region of C57BL/6 mice (19‐21 g, male). On days 0, 5, 10, 14, and 21 after implantation, mice were euthanized by inhalation of isoflurane. The skin tissues around the implantation site were incised and the hydrogels were observed and recorded using a digital camera. Finally, the hydrogels were taken out and weighed after lyophilization.

*In Vivo Biocompatibility*: In vivo biocompatibility was evaluated by implanting PZBA‐EGCG‐ALC Janus hydrogels into the Achilles tendon site of SD rats (200–260 g, male). After 28 days of implantation, major organ tissues were collected and analyzed by H&E staining.

### In Vivo Rat Liver Defect Repair

For the in vivo rat liver defect repair model, rats were anesthetized using isoflurane (2–3% isoflurane in oxygen) in an anesthetizing chamber. Anesthesia was maintained using a nose cone. Prior to surgery the rats were placed on surgical pads. The liver was exposed by laparotomy. A 5 mm in length and 2 mm in depth wound was made in the liver using surgical scalpel. Subsequently, a piece of PZBA‐EGCG‐ALC Janus hydrogel ≈2 cm by 3 cm was placed to seal the hemorrhage defect and gently pressed for 10 s. At the end of the experiment, the rats were euthanized by CO_2_ inhalation. The liver defect repair model was performed on two independent samples with similar results.

### In Vivo Rat Femoral Artery Defect Repair

For the in vivo rat femoral artery defect repair model, rats were anesthetized using isoflurane (2≈3% isoflurane in oxygen) in an anesthetizing chamber. Anesthesia was maintained using a nose cone. Prior to surgery, the rats were placed on surgical pads. The femoral artery is exposed through a thigh incision. Using a 4.5# needle, make a puncture in the femoral artery to create a wound approximately 2 mm in diameter. Subsequently, a piece of PZBA‐EGCG‐ALC Janus hydrogel about 2 cm by 3 cm was placed to seal the hemorrhage defect and gently pressed for 10 seconds. At the end of the experiment, the rats were euthanized by CO_2_ inhalation. The femoral artery defect repair model was performed on two independent samples with similar results.

### Rat Achilles Adhesion Model

All experimental animals were treated in accordance with the policies of Zhejiang University. Twenty‐five male 12‐week Sprague‐Dawley rats with body weights of 200–300 g were randomly grouped into 5 clusters defined as blank (without any treatment), non‐suture (partially‐ruptured tendons without any treatment), suture (partially‐ruptured tendons repaired with suture), ALC‐1000 (Janus hydrogel without PZBA‐EGCG prodrug macromolecules), PZBA‐EGCG_3_‐ALC‐1500 (non‐Janus hydrogel with PZBA‐EGCG prodrug macromolecules), and PZBA‐EGCG_3_‐ALC‐1000 (Janus hydrogel with PZBA‐EGCG prodrug macromolecules), respectively. The rats were anesthetized by administering an intravenous injection of 3% pentobarbital sodium at a dosage of 30 mg kg^−1^. After skin preparation and sterilization, a 2 cm midline incision was created at the rat tendon site. Next, the tendon was transected ≈5 mm from the talocalcaneal tuberosity and then sutured with 6‐0 silk suture using a modified Kessler technique. Subsequently, the hydrogels encircled the surgical area. The incision was then closed using sutures.

### Macroscopic Evaluation and Peritendinous Adhesion Scoring

On the 28th day, the biological status of the surgical site (e.g., inflammation, infection, and ulceration) was evaluated. The rats were then sacrificed to expose their status of tendon healing. During surgical observation, the severity of tendon adhesions was macroscopically evaluated according to a developed adhesion scoring system divided into five grades:^[^ ^46^ ^]^ Grade 1, little or no adhesions were scanned; Grade 2, only limited adhesions were observed at the surgical site, which could be separated by blunt dissection; Grade 3, a moderate amount of adherent tissues were formed, encompassing ≤50% of the tendon, which could be separated by sharp dissection; Grade 4, a large amount of adhesion tissues were formed, encompassing 51%‐97.5% of the tendon, which could still be separated by sharp dissection; Grade 5, more than 97.5% of the tendon was covered by a large amount of adhesion tissues, which were almost impossible to be separated by sharp dissection. All samples were scored in parallel by two independent observers and grouping information was masked.

### Two‐Photon Microscopy

Healed tendons from each group were imaged under two‐photon microscopy using an excitation wavelength of 850 nm. Image acquisition employed a resolution of 800 pixels × 800 pixels, light collection through a 420–460 nm filter, and a pixel acquisition rate of 4 µm per pixel. Collagen fiber second harmonic generation (SHG) fluorescence in both injured and uninjured regions was observed. 3D reconstructions of the elastic fiber network from the full image stack with a stack step of 10 µm per slice and a number of stacked full images of 75 slices. Qualitative analysis was conducted to assess collagen fiber density and arrangement. Quantitative analysis was performed by integrating the fluorescence intensity of each image using Image J software.

### Histology Assessment

On the 14th and 28th day, regenerated tendons and adjacent muscles were taken and fixed in 4% paraformaldehyde solution for 24 h. The fixed tissues were then stained with hematoxylin and eosin (H&E) and Masson's trichrome, followed with examination under light microscope (Nikon, Japan).

Histologic assessment of tendon adhesions was scored from 1 to 4 according to the following scoring system:^[^ ^47^ ^]^ Score 1, no adhesions; Score 2, mild adhesions (<33% of the repaired tendon surface); Score 3, moderate adhesions (33%≈66% of the repaired tendon surface); Score 4, severe adhesions (>66% of the repaired tendon surface).

The status of tendon healing was further assessed using the following scoring system:^[^ ^47^, ^48^ ^]^ Score 1, excellent (good continuity and smooth epitendon surface); Score 2, good (well‐structured intratendinous collagen bundles and disrupted epitendon by adhesions); Score 3, fair (poorly‐structured intratendinous collagen bundles and partially ruptured); Score 4, poor (failure of tendon healing and extensive granulation tissue formation). The histopathologic assessment of the inflammatory response was further performed by examining the number and aggregation of inflammatory cells such as macrophages, lymphocytes, neutrophils, and mast cells.^[^ ^47^, ^48^ ^]^ Inflammation was semi‐quantified using a score from 1 to 4: Score 1, only sporadic inflammatory cells were observed; Score 2, mild inflammatory cell infiltration; Score 3, moderate inflammatory cell infiltration; Score 4, severe inflammatory cell infiltration. All samples were scored in parallel by two independent observers based on the 4 fields of view of their histologic sections, and grouping information was masked.

### Real‐Time Polymerase Chain Reaction (qPCR) Analysis

On the 14th and 28th day, the total RNAs of collected tendons were extracted and purified using a Tissue RNA Purification Kit PLUS (EZBioscience, USA), and then were reverse‐transcribed to cDNA. The cDNA and gene primer sequences were obtained by qPCR to detect the expression of TNF‐α, HIF‐α, IL‐1β, IL‐6, SOD‐1, SOD‐2, TGF‐β1, COL‐1, and COL‐3 (Table S6). Glyceraldehyde 3‐phosphate dehydrogenase (GAPDH) was used as a housekeeping gene. The sequences of the primers are listed in Table S6. The mRNA levels were quantified using the CFX96 (Bio‐Rad, Hercules, CA, USA).

### Assessment of Biomechanical Properties of Regenerated Tendons

Biomechanical properties of the regenerated tendon tissues were performed by a mechanical testing machine (Instron‐2 kN). The tensile rate was fixed at 10 mm min^−1^ at room temperature. Load and displacement data were collected during the experiment. All the tests were repeated three times for each group.

### Statistical Analysis

The data in this research were analyzed using Origin 2022 software and were reported as the means ± standard deviation (SD). The statistical significance was determined using One‐way ANOVA and Student's *t*‐test, with a significance level set at *p* < 0.05.

## Conflict of Interest

The authors declare no conflict of interest.

## Author Contributions

C.O. and T.T. contributed equally to this work. H.Y., L.W., and H.L. conceived the project. C.O. designed and analyzed the experiments, and wrote the manuscript. Z.N., J.Y., Y.D., X.Z., W.Z., and Z.L. assisted with in vivo experiments and analysis. J.L., D.C., Y.W., X.W., H.Y., and X.Y. assisted with the synthesis of polymer and the characterizations of the hydrogels. T.T. assisted with qPCR and cell experiments.

## Supporting information



Supporting Information

Supplemental Movie 1

Supplemental Movie 2

## Data Availability

The data that support the findings of this study are available from the corresponding author upon reasonable request.
