# Supplementary material for: One‐Step Formed Janus Hydrogel with Time‐Space Regulating Properties for Suture‐Free and High‐Quality Tendon Healing
Source: Adv Sci (Weinh). 2025 Feb 8;12(13):2411400. doi: 10.1002/advs.202411400 (PMC11967842; doi:10.1002/advs.202411400)
Supplement: Supplementary file 1 — Supporting Information [file ADVS-12-2411400-s003.docx]

**Supporting Information for**

**One-Step Formed Janus Hydrogel with Time-Space Regulating Properties for Suture-Free and High-Quality Tendon Healing**

Chenguang Ouyang^1^, Tian Tu^2^, Haojie Yu^1*^, Li Wang^1*^, Zhipeng Ni^1^, Jian Yang^1^, Yanzhao Dong^3^, Xiaodi Zou^3^, Weijie Zhou^3^, Jinyi Liu^1^, Dingning Chen^1^, Yu Wang^1^, Xudong Wu^1^, Hong Yi^1^, Xunchun Yuan^1^, Zhenfeng Liu^4^, Hui Lu^3*^

1 State Key Laboratory of Chemical Engineering, College of Chemical and Biological Engineering, Zhejiang University, Hangzhou 310058, Zhejiang Province, China.

2 Department of plastic and aesthetic, the First Affiliated Hospital, College of Medicine, Zhejiang University, Hangzhou 310003, Zhejiang Province, China.

3 Department of Orthopedics, the First Affiliated Hospital, College of Medicine, Zhejiang University, Hangzhou 310003, Zhejiang Province, China.

4 Department of Nuclear Medicine, The First Affiliated Hospital, College of Medicine, Zhejiang University, Hangzhou 310003, Zhejiang Province, China.

*E-mail: hjyu@zju.edu.cn (Haojie Yu); opl_wl@dial.zju.edu.cn (Li Wang); huilu@zju.edu.cn (Hui Lu)


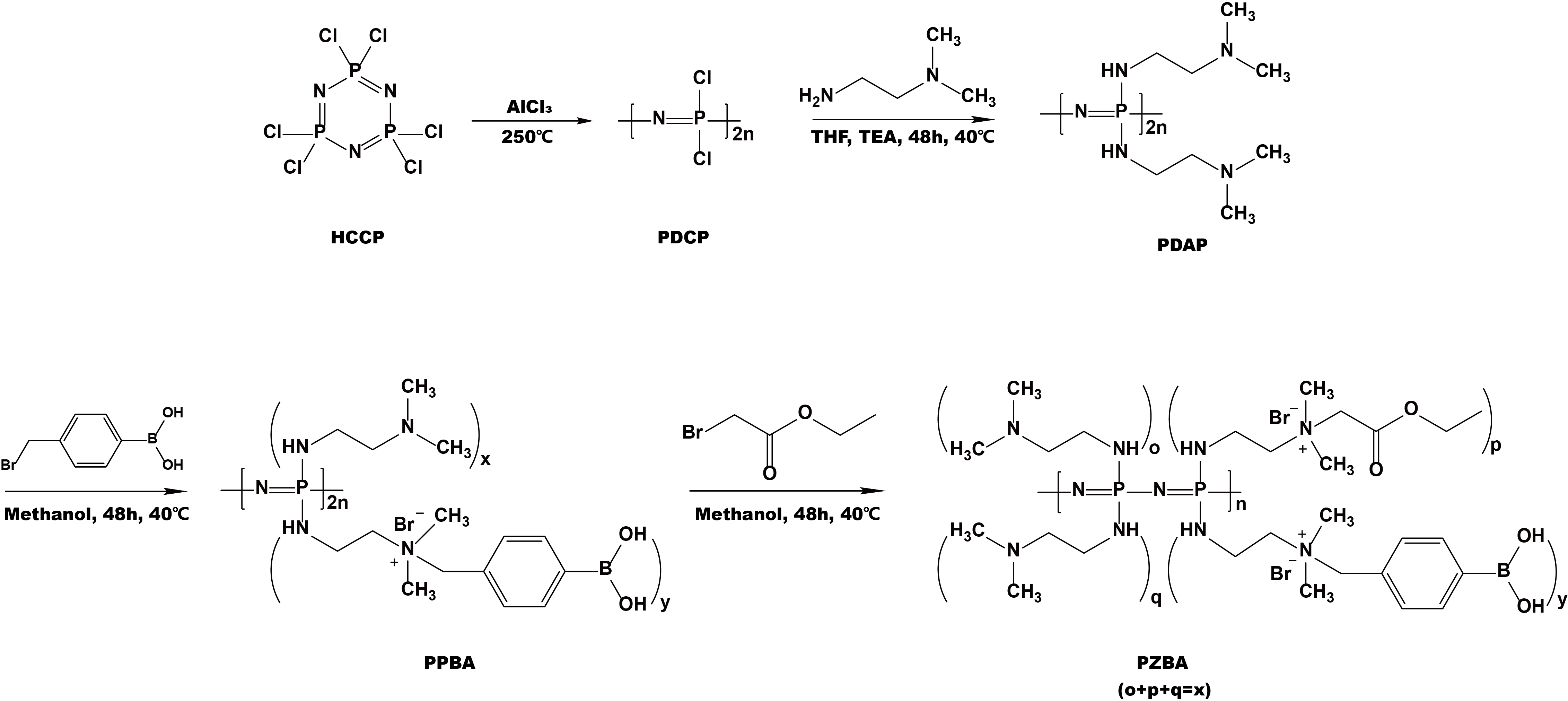


**Figure S1**. The synthesis route of PZBA.


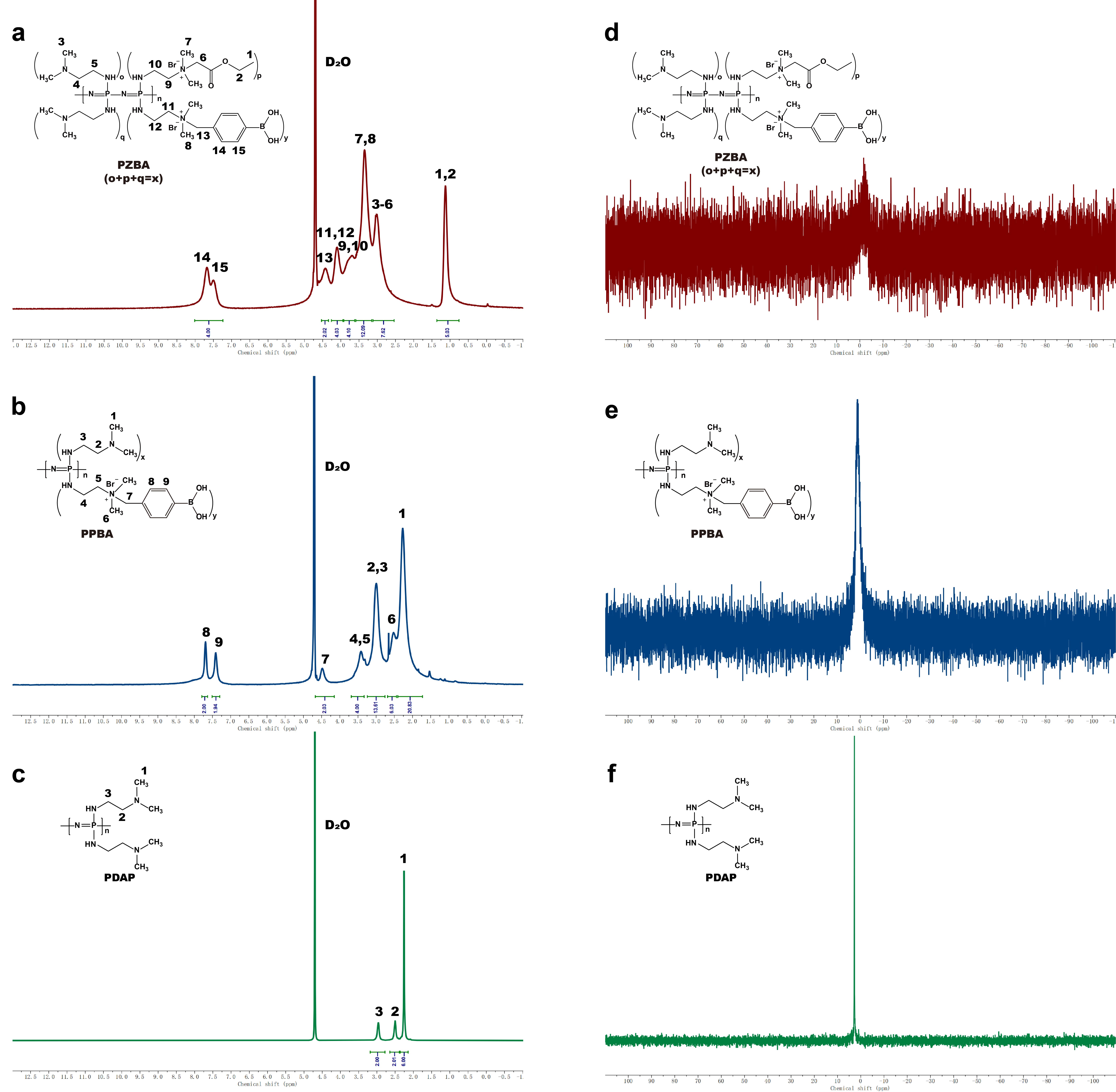


**Figure S2**. NMR spectra of PDAP, PPBA and PZBA. a-c) ^1^H NMR spectra of PDAP, PPBA and PZBA. e-f) ^31^P NMR spectra of PDAP, PPBA and PZBA.


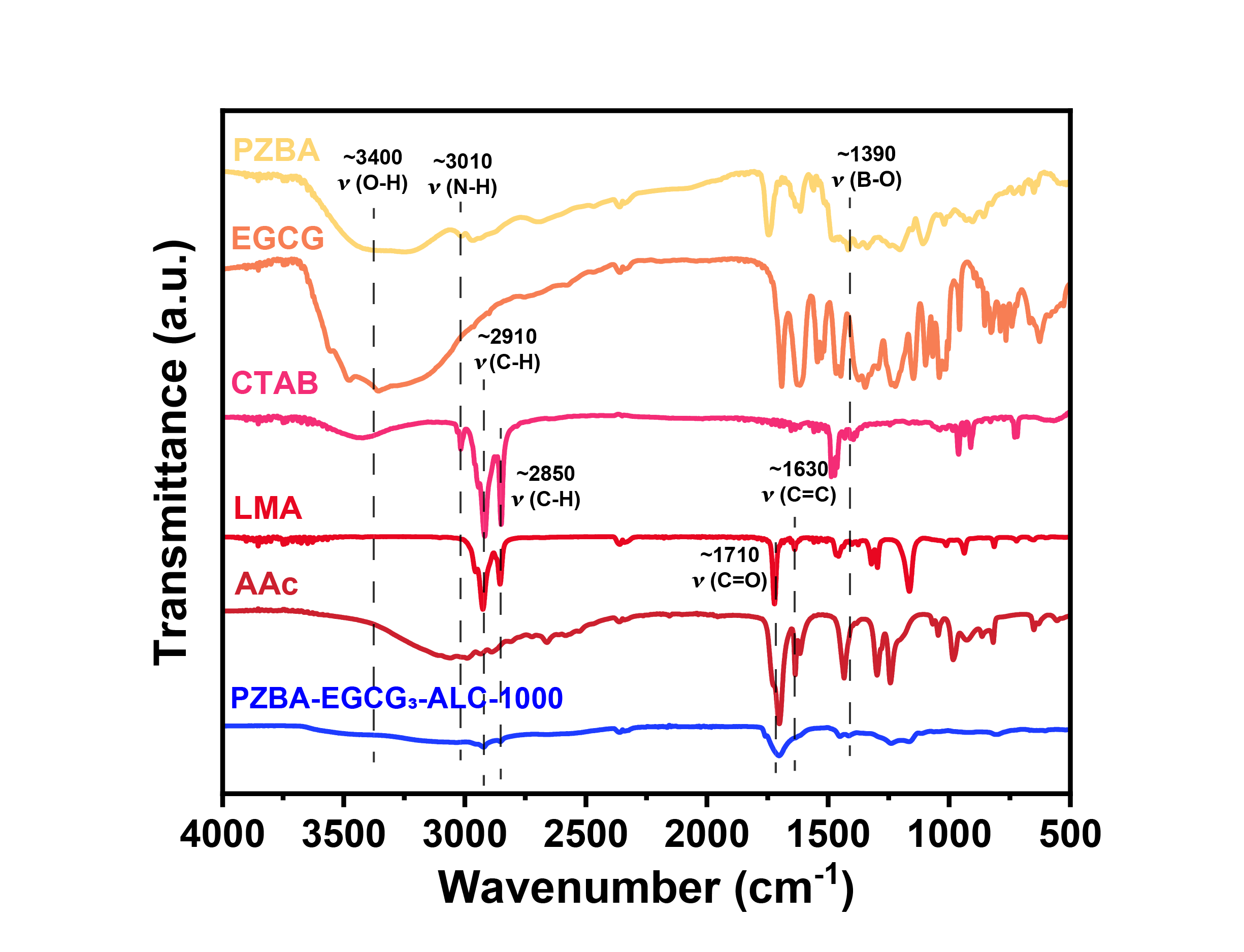


**Figure S3**. FT-IR spectra of AAc, LMA, CTAB, PZBA, EGCG, ALC-1000 and PZBA-EGCG_3_-ALC-1000 hydrogels.


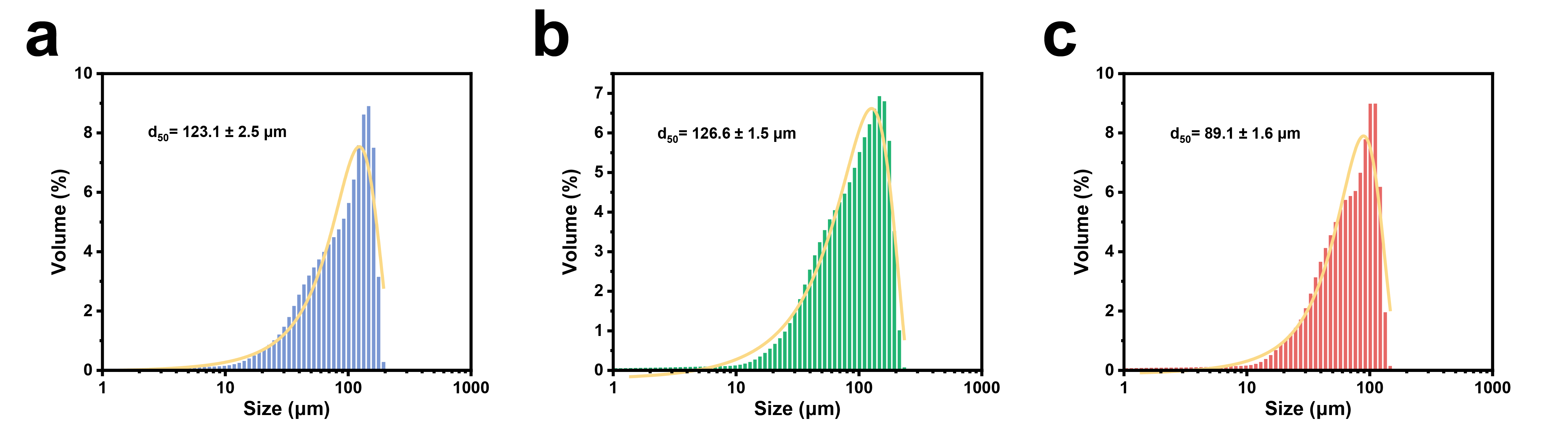


**Figure S4**. Stability of the particle size of (a) PZBA-EGCG_3_-ALC-500, (b) PZBA-EGCG_3_-ALC-1000, (c) PZBA-EGCG_3_-ALC-1500 hydrogel precursor emulsions after 24 h placement.


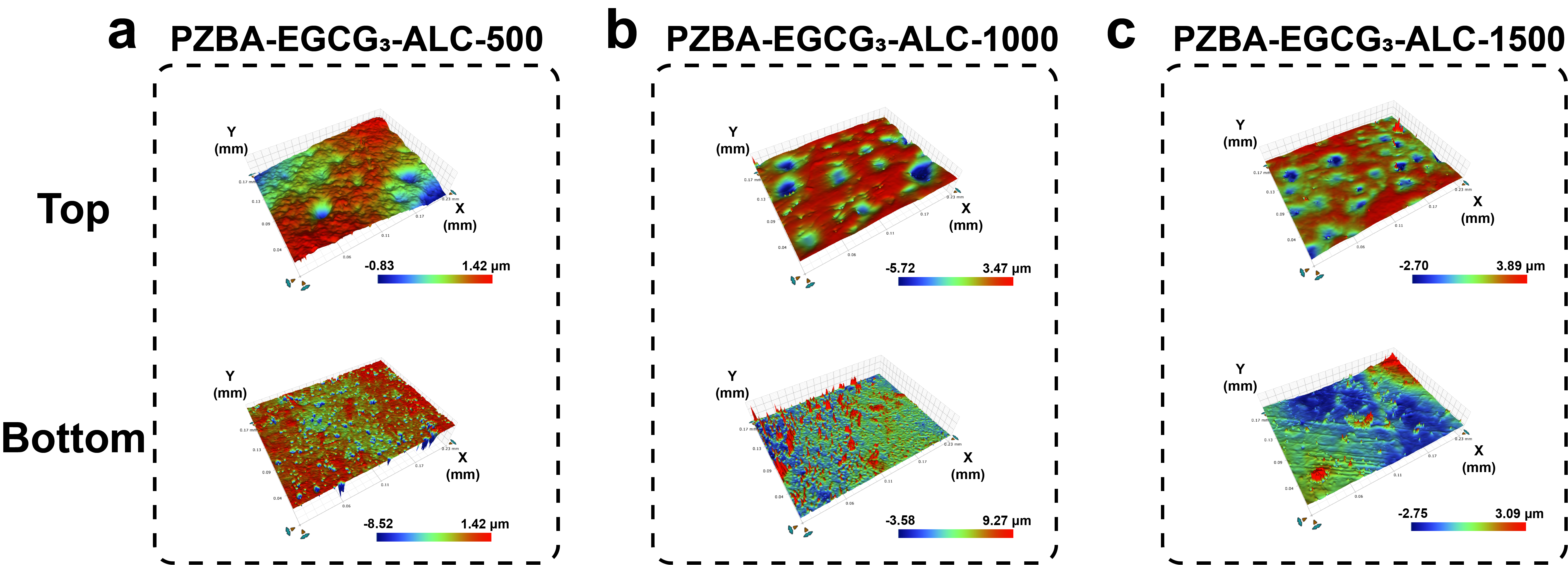


**Figure S5**. The 3D contour images of the top and bottom surface of (a) PZBA-EGCG_3_-ALC-500, (b) PZBA-EGCG_3_-ALC-1000, (c) PZBA-EGCG_3_-ALC-1500 hydrogels.


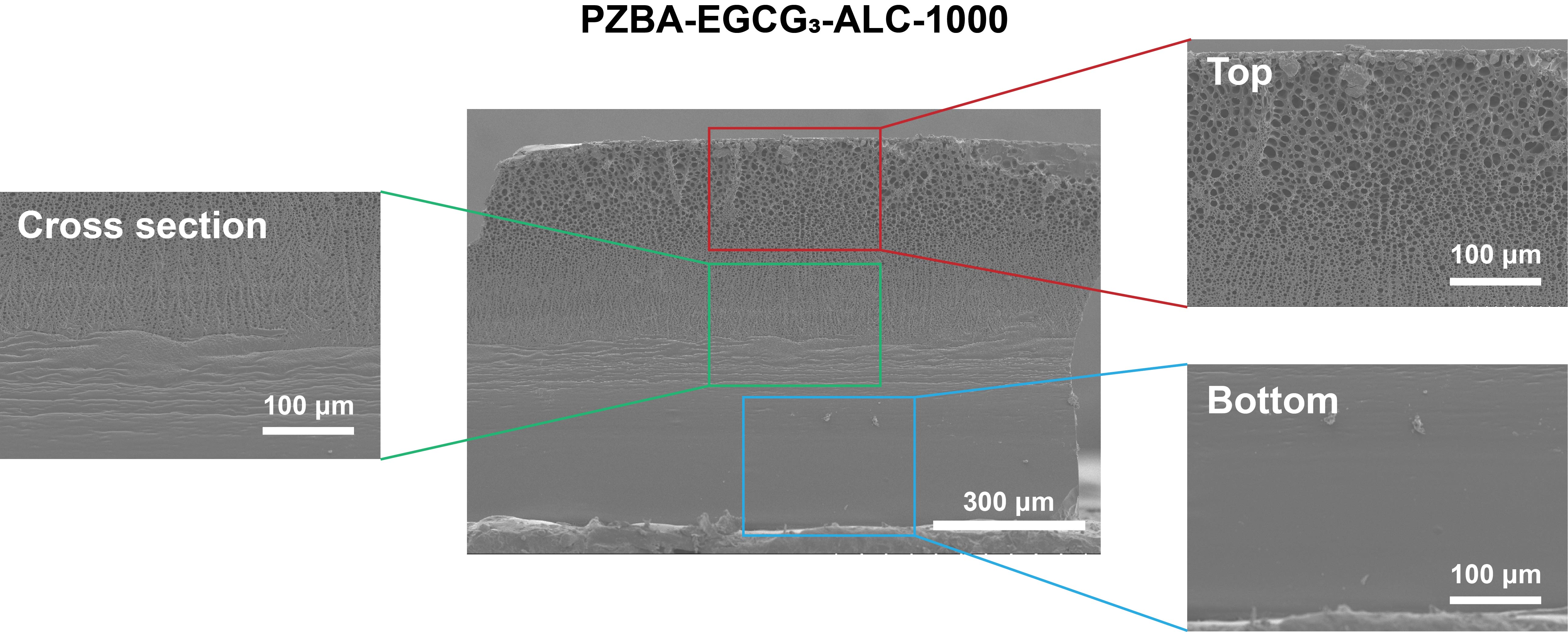


**Figure S6.** The SEM pictures of the section morphology for PZBA-EGCG_3_-ALC-1000 hydrogel.


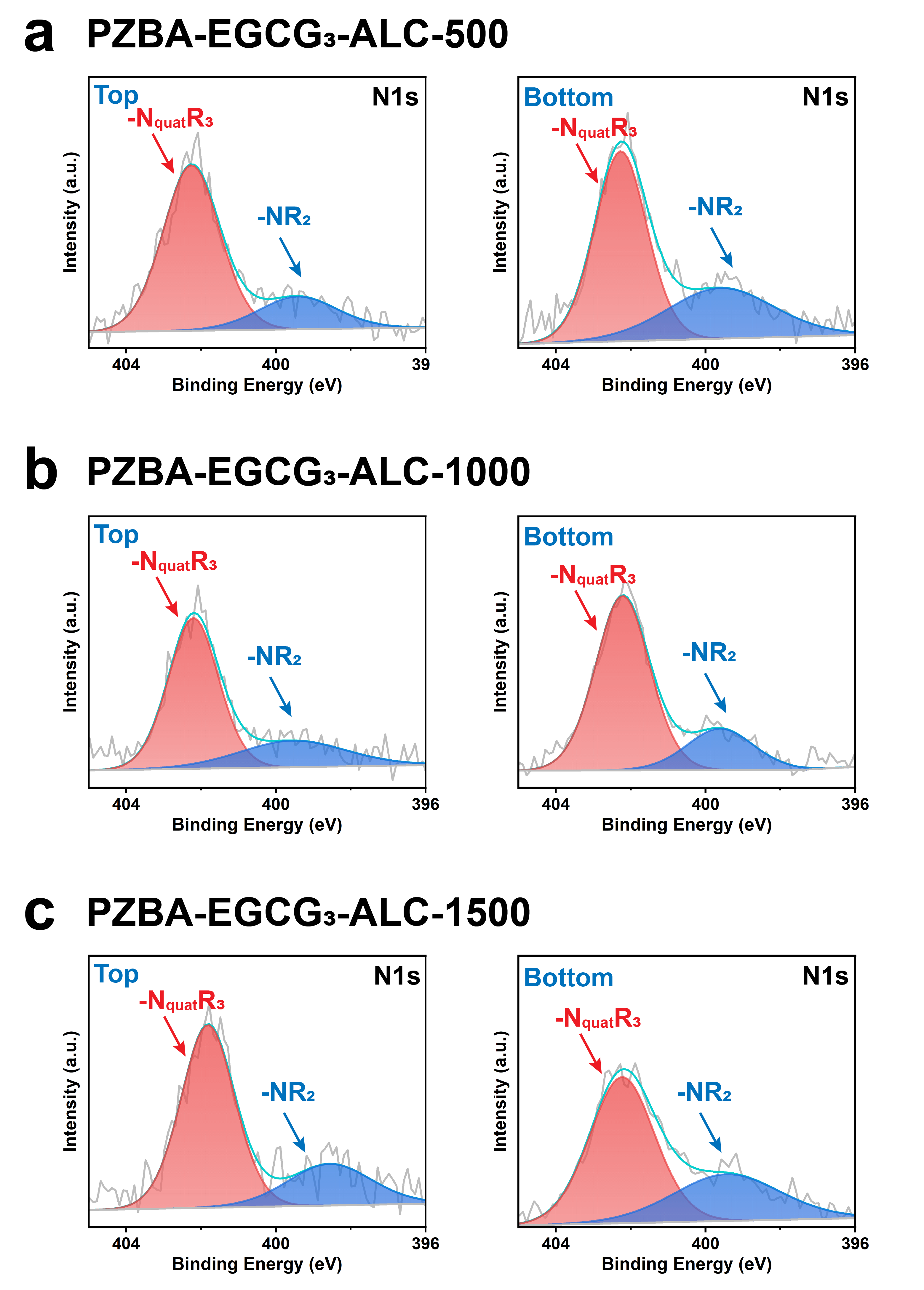


**Figure S7.** Peak-fitting XPS spectra in the N1s regions of the top surface and bottom surface for (a) PZBA-EGCG_3_-ALC-500, (b) PZBA-EGCG_3_-ALC-1000, (c) PZBA-EGCG_3_-ALC-1500 hydrogels.


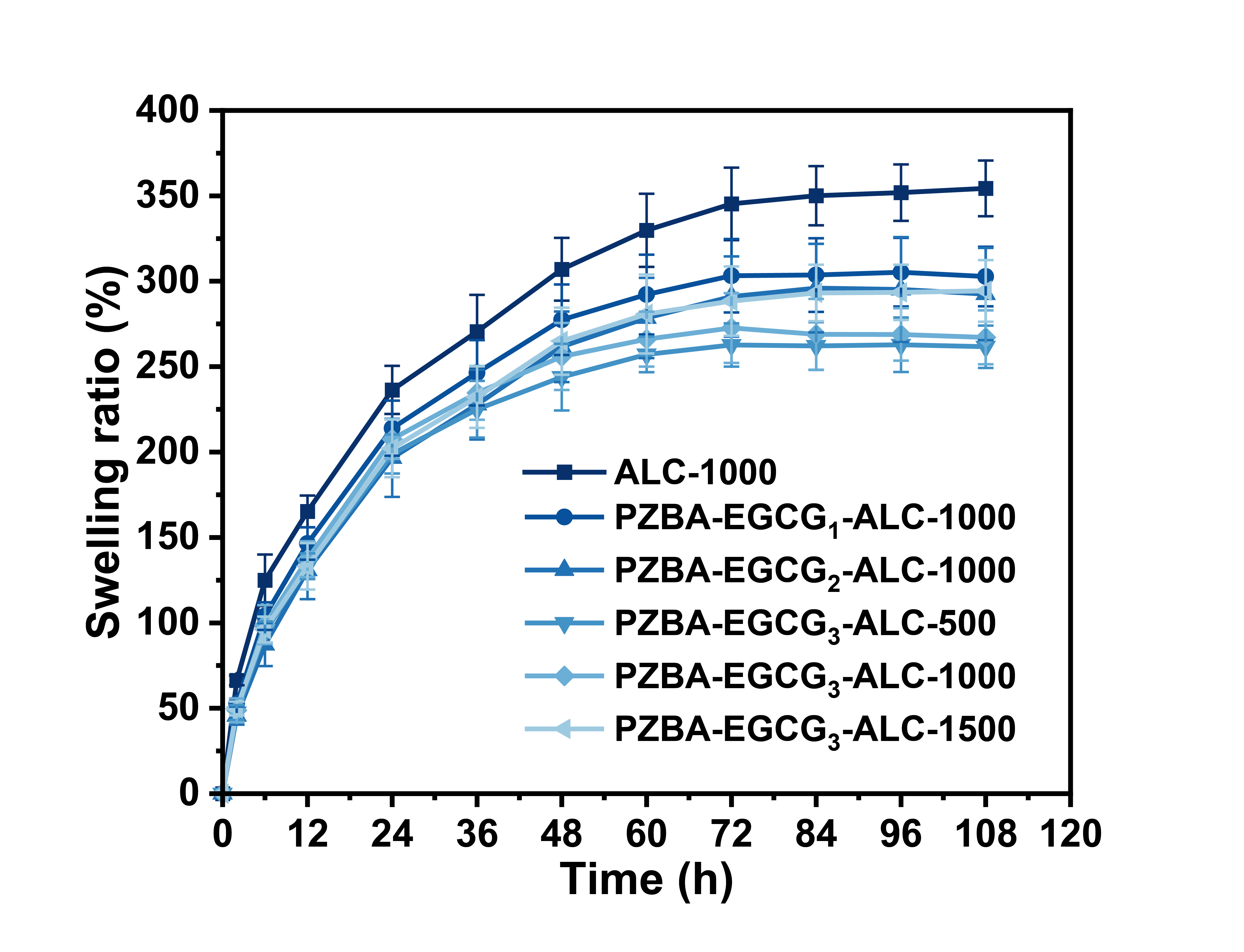


**Figure S8.** Swelling rate of ALC-1000, PZBA-EGCG_1_-ALC-1000, PZBA-EGCG_2_-ALC-1000, PZBA-EGCG_3_-ALC-500, PZBA-EGCG_3_-ALC-1000 and PZBA-EGCG_3_-ALC-1500 hydrogels in PBS at different time (37 °C, pH = 7.4).


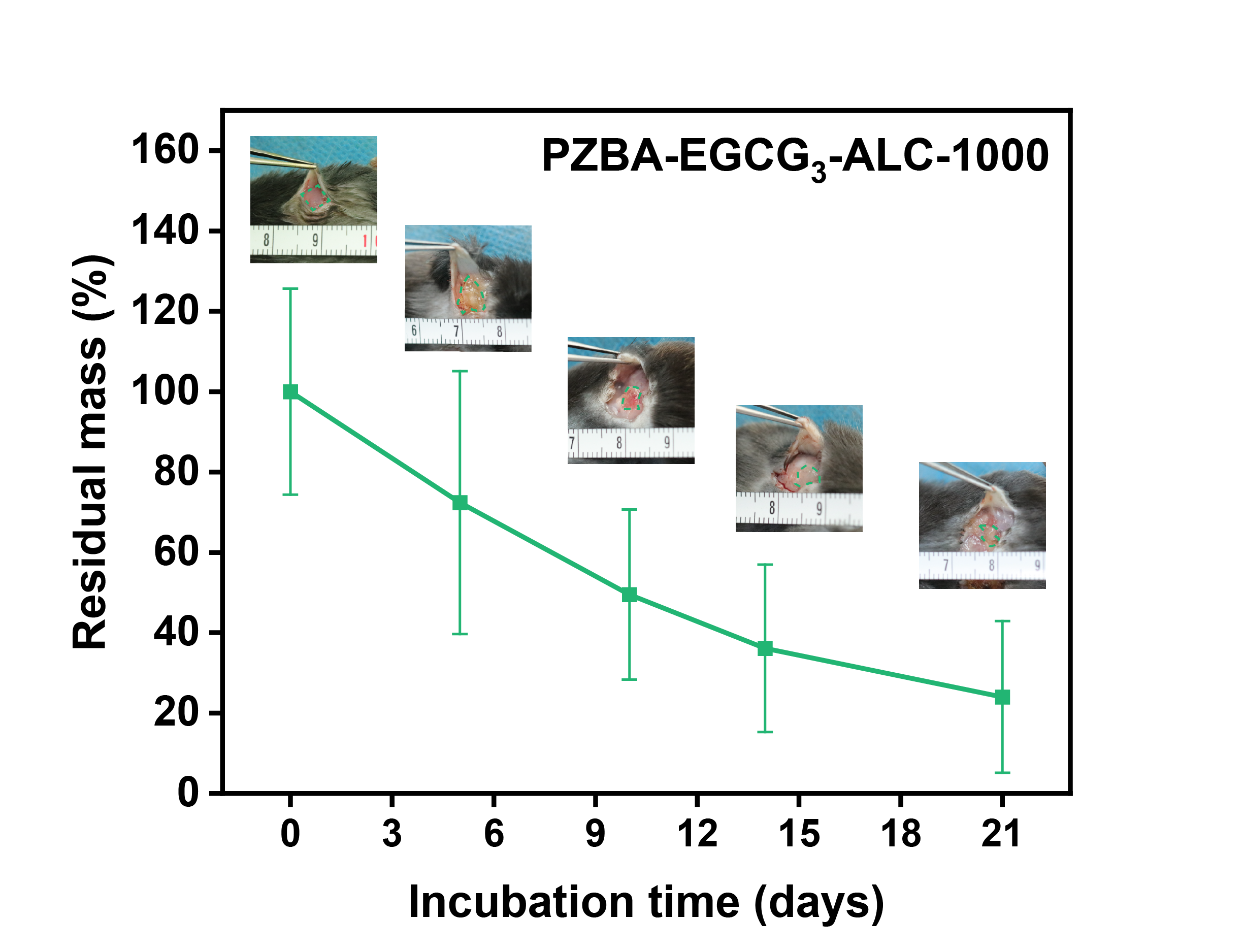


**Figure S9.** Residual mass percentage and digital photographs (inset pictures) of PZBA-EGCG-ALC Janus hydrogels after subcutaneous implantation in mice for 5, 10, 14, and 21 days.


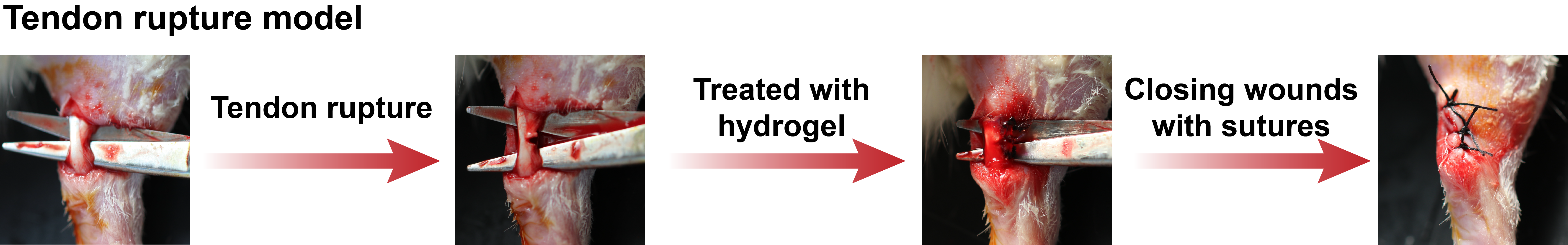


**Figure S10.** Surgical operation process showing that the surgical site of the tendon can be easily wrapped with the PZBA-EGCG-ALC Janus hydrogel, with the more adhesive side facing the injury.


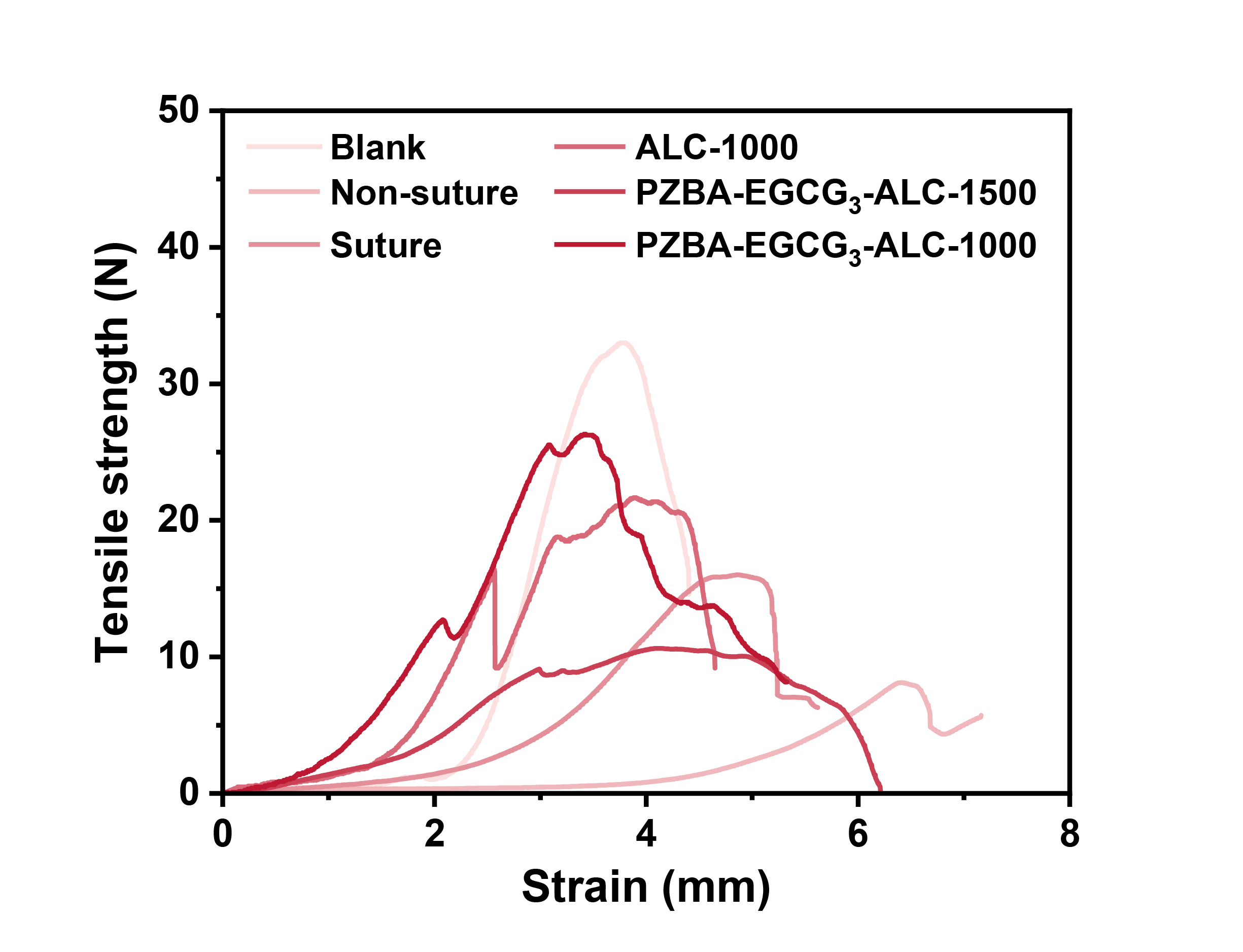


**Figure S11.** The representative load-to-failure curves of the healed tendons on the 14th day.


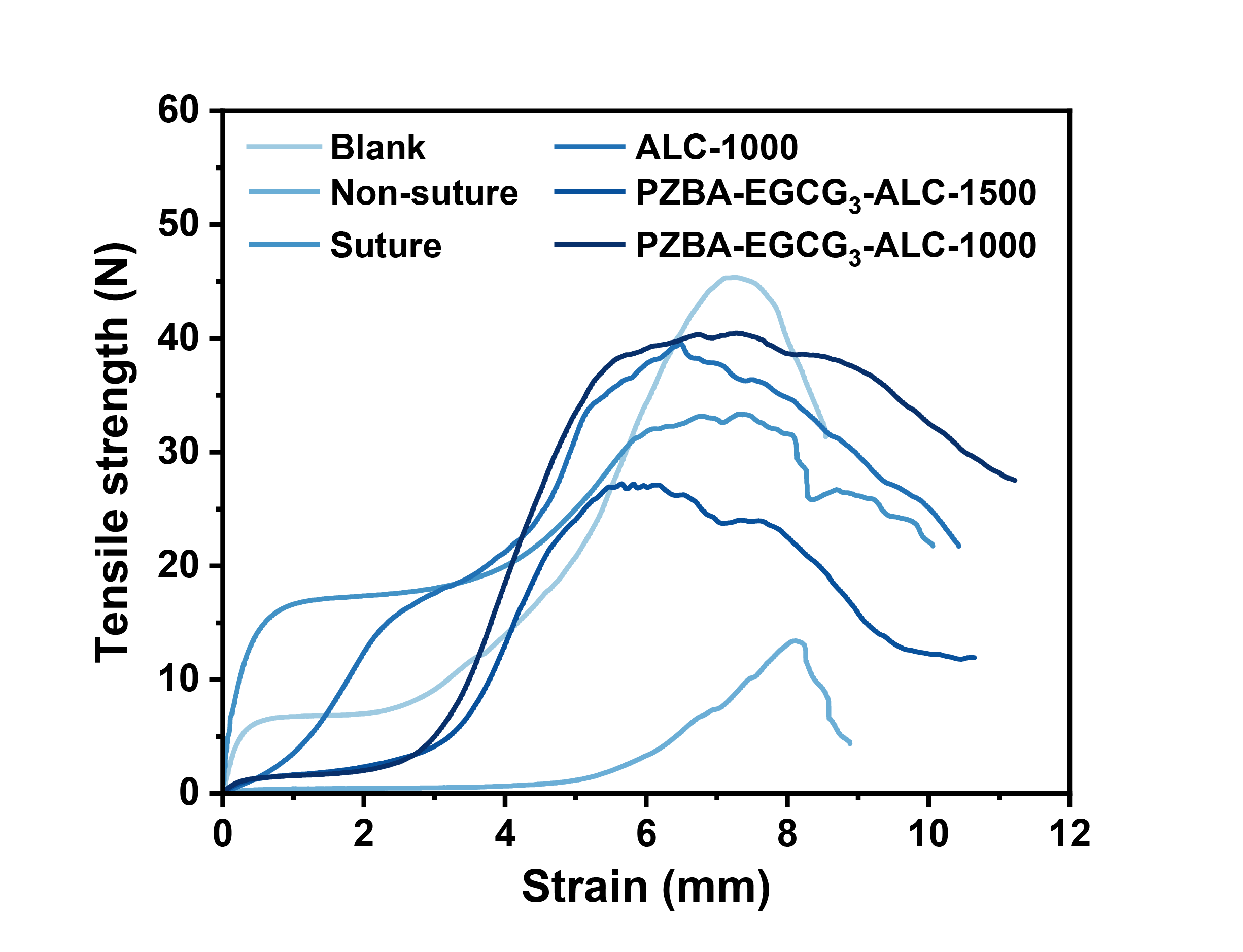


**Figure S12.** The representative load-to-failure curves of the healed tendons on the 28th day.

**
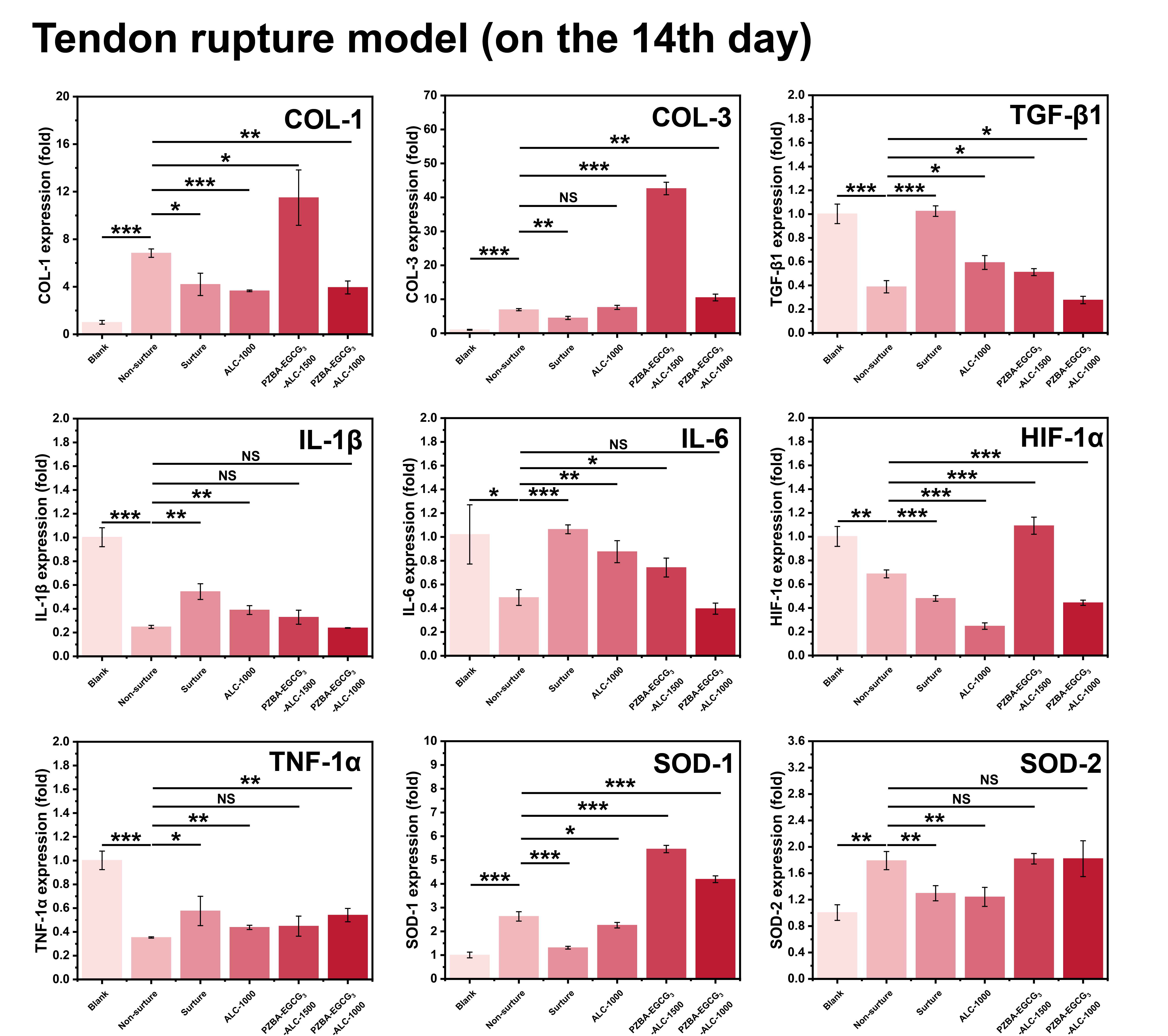
**

**Figure S13.** The COL-1, COL-3, TGF-β1, IL-1β, IL-6, HIF-1α, TNF-α, SOD-1, and SOD-2 gene expressions of Achilles tendons on the 14th day. Values and error bars in Figure S12 represent the mean and standard deviation (*n* = 3 independent samples). Statistical significance and *p* values were determined using a two-tailed Student’s t-test with unequal variance: NS *p* > 0.05; * *p* ≤ 0.05; ** *p* ≤ 0.01; *** *p* ≤ 0.001.


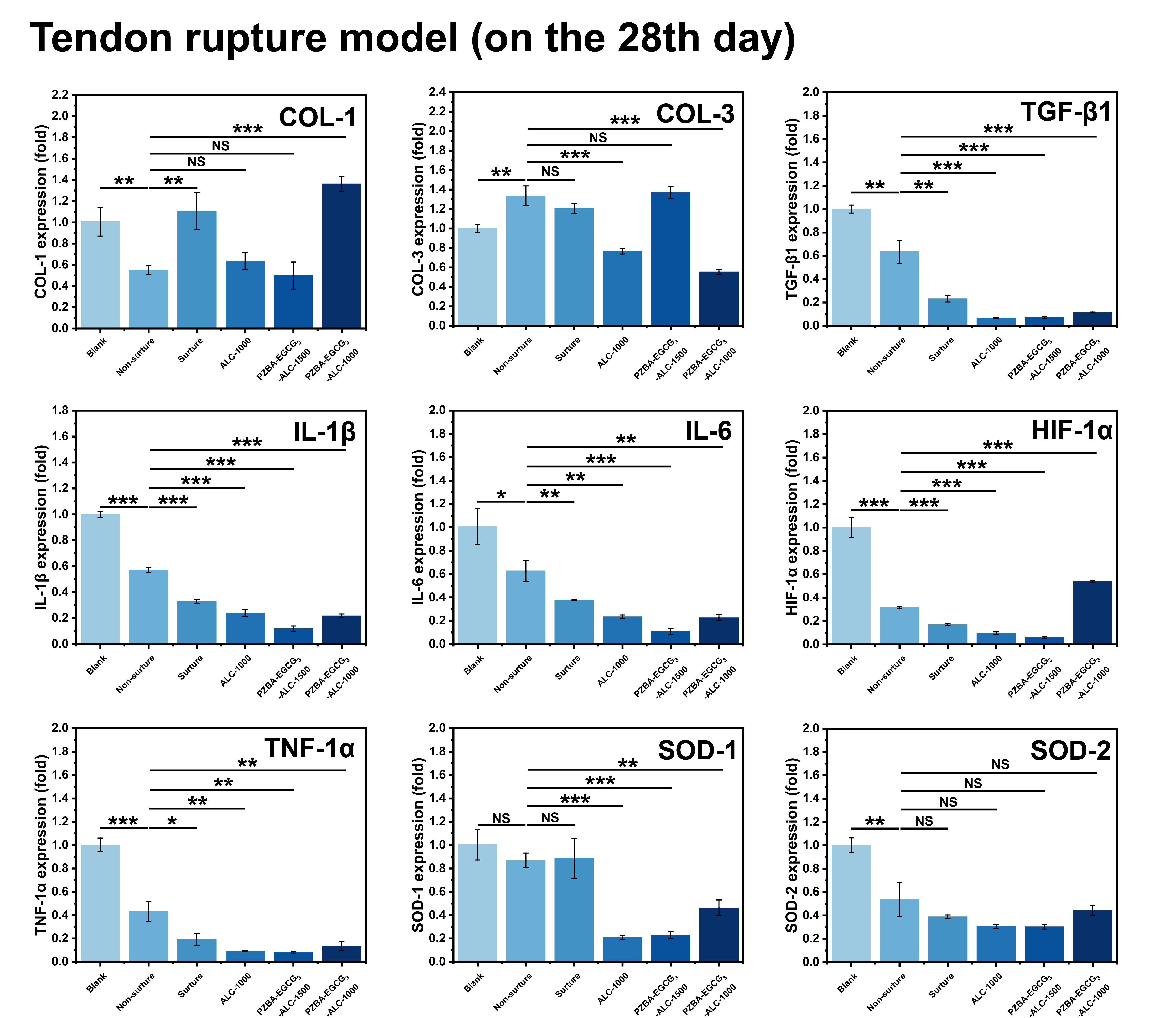


**Figure S14.** The COL-1, COL-3, TGF-β1, IL-1β, IL-6, HIF-1α, TNF-α, SOD-1, and SOD-2 gene expressions of Achilles tendons on the 28th day. Values and error bars in Figure S12 represent the mean and standard deviation (*n* = 3 independent samples). Statistical significance and *p* values were determined using a two-tailed Student’s t-test with unequal variance: NS *p* > 0.05; * *p* ≤ 0.05; ** *p* ≤ 0.01; *** *p* ≤ 0.001.

**Table S1.** The formulations of PZBA-EGCG-ALC hydrogels.

| **Sample** |  | **PZBA** | |  | **EGCG** | |  | **AAc** | |  | **LMA** | |  | **CTAB** | |  | **LAP** | |  | **H_2_O** |  | **Stirring speed** |  | ***t*** |  | ***T*** |
| --- | --- | --- | --- | --- | --- | --- | --- | --- | --- | --- | --- | --- | --- | --- | --- | --- | --- | --- | --- | --- | --- | --- | --- | --- | --- | --- |
|  |  | **g** | **mmol**  **(unit)** |  | **g** | **mmol** |  | **g** | **mmol** |  | **g** | **mmol** |  | **g** | **mmol** |  | **g** | **mmol** |  | **mL** |  | **RPM** |  | **h** |  | **℃** |
| **ALC-1000** |  | - | - |  | - | - |  | 4.3933 | 61.06 |  | 0.1881 | 0.71 |  | 0.3826 | 1.04 |  | 0.0069 | 0.02 |  | 5.0 |  | 1000 |  | 4 |  | 40 |
| **PZBA-EGCG_3_-ALC-500** |  | 0.3022 | 0.60 |  | 0.0684 | 0.15 |  | 4.4651 | 61.06 |  | 0.1856 | 0.71 |  | 0.3827 | 1.04 |  | 0.0067 | 0.02 |  | 5.0 |  | 500 |  | 4 |  | 40 |
| **PZBA-EGCG_1_-ALC-1000** |  | 0.3095 | 0.60 |  | 0.0231 | 0.05 |  | 4.3987 | 61.06 |  | 0.1885 | 0.71 |  | 0.3822 | 1.04 |  | 0.0066 | 0.02 |  | 5.0 |  | 1000 |  | 4 |  | 40 |
| **PZBA-EGCG_2_-ALC-1000** |  | 0.3124 | 0.60 |  | 0.0458 | 0.10 |  | 4.5021 | 61.06 |  | 0.1779 | 0.71 |  | 0.3801 | 1.04 |  | 0.0072 | 0.02 |  | 5.0 |  | 1000 |  | 4 |  | 40 |
| **PZBA-EGCG_3_-ALC-1000** |  | 0.2988 | 0.60 |  | 0.0679 | 0.15 |  | 4.4282 | 61.06 |  | 0.1763 | 0.71 |  | 0.3749 | 1.04 |  | 0.0068 | 0.02 |  | 5.0 |  | 1000 |  | 4 |  | 40 |
| **PZBA-EGCG_3_-ALC-1500** |  | 0.2944 | 0.60 |  | 0.0677 | 0.15 |  | 4.4673 | 61.06 |  | 0.1902 | 0.71 |  | 0.3901 | 1.04 |  | 0.0069 | 0.02 |  | 5.0 |  | 1500 |  | 4 |  | 40 |

**Table S2.** Tensile stress, strain and toughness of PZBA-EGCG-ALC hydrogels with different stirring speeds.

| **Sample** |  | **Stress (MPa)** |  | **Strain (%)** |
| --- | --- | --- | --- | --- |
| PZBA-EGCG_3_-ALC-500 |  | 0.35 ± 0.04 |  | 897.67 ± 51.44 |
| PZBA-EGCG_3_-ALC-1000 |  | 0.51 ± 0.04 |  | 922.89 ± 28.59 |
| PZBA-EGCG_3_-ALC-1500 |  | 0.25 ± 0.06 |  | 929.48 ± 52.78 |

**Table S3.** Tensile stress, strain and toughness of PZBA-EGCG-ALC hydrogels with different EGCG amounts at 1000 RPM.

| **Sample** |  | **Stress (MPa)** |  | **Strain (%)** |
| --- | --- | --- | --- | --- |
| ALC-1000 |  | 0.34 ± 0.20 |  | 817.63 ± 70.57 |
| PZBA-EGCG_1_-ALC-1000 |  | 0.62 ± 0.02 |  | 853.00 ± 57.35 |
| PZBA-EGCG_2_-ALC-1000 |  | 0.67 ± 0.05 |  | 913.78 ± 51.79 |
| PZBA-EGCG_3_-ALC-1000 |  | 0.51 ± 0.04 |  | 922.89 ± 28.59 |

**Table S4.** Compressive stress and strain of PZBA-EGCG-ALC hydrogels with different stirring speeds.

| **Sample** |  | **Stress (MPa)** |  | **Strain (%)** |
| --- | --- | --- | --- | --- |
| PZBA-EGCG_3_-ALC-500 |  | 4.98 ± 2.62 |  | 90.00 ± 0.00 |
| PZBA-EGCG_3_-ALC-1000 |  | 8.63 ± 6.21 |  | 89.72 ± 0.47 |
| PZBA-EGCG_3_-ALC-1500 |  | 11.95 ± 4.30 |  | 89.94 ± 0.09 |

**Table S5.** Compressive stress and strain of PZBA-EGCG-ALC hydrogels with different EGCG amounts at 1000 RPM.

| **Sample** |  | **Stress (MPa)** |  | **Strain (%)** |
| --- | --- | --- | --- | --- |
| ALC-1000 |  | 5.84 ± 0.68 |  | 90.00 ± 0.00 |
| PZBA-EGCG_1_-ALC-1000 |  | 3.69 ± 2.08 |  | 89.19 ± 1.41 |
| PZBA-EGCG_2_-ALC-1000 |  | 6.03 ± 2.92 |  | 90.00 ± 0.00 |
| PZBA-EGCG_3_-ALC-1000 |  | 8.63 ± 6.21 |  | 89.72 ± 0.47 |

**Table S6.** Nucleotide primers used for RT-PCR.

| **Abbreviations** |  | **Primer sequence (5’-3’)** |
| --- | --- | --- |
| **GAPDH** |  | Forward: GAGCGAGATCCCGTCAAGATCAAA |
|  |  | Reverse: CACAGTCTTCTGAGTGGCAGTGAT |
| **COL-1** |  | Forward: GGATCGACCCTAACCAAGGC |
|  |  | Reverse: ATCGGAACCTTCGCTTCCA |
| **COL-3** |  | Forward: GAAGGGCAGGGAACAACTGA |
|  |  | Reverse: GGGCAGTCTAGTGGCTCATC |
| **IL-1β** |  | Forward: GGGATGATGACGACCTGCTA |
|  |  | Reverse: TGTCGTTGCTTGTCTCTCCT |
| **IL-6** |  | Forward: CCACTGCCTTCCCTACTTCA |
|  |  | Reverse: TTCTGACAGTGCATCATCGC |
| **TGF-β1** |  | Forward: TCGCTTTGTACAACAGCACC |
|  |  | Reverse: ACTGCTTCCCGAATGTCTGA |
| **TNF-α** |  | Forward: CGTCGTAGCAAACCACCAAG |
|  |  | Reverse: GAGGCTGACTTTCTCCTGGT |
| **HIF-α** |  | Forward: TGCTTGGTGCTGATTTGTGA |
|  |  | Reverse: GGTCAGATGATCAGAGTCCA |
| **SOD-1** |  | Forward: CACTTCGAGCAGAAGGCAAG |
|  |  | Reverse: CCAACATGCCTCTCTTCATC |
| **SOD-2** |  | Forward: CACATTAACGCGCAGATCATG |
|  |  | Reverse: CCTTAGGGCTCAGGTTTGTC |

**Movie S1.** Sealing of an *in vivo* rat liver using the PZBA-EGCG_3_-ALC-1000 hydrogel.

**Movie S2.** Sealing of an *in vivo* rat femoral artery using the PZBA-EGCG_3_-ALC-1000 hydrogel.
